# Estimating Relative Changes of Metabolic Fluxes

**DOI:** 10.1371/journal.pcbi.1003958

**Published:** 2014-11-20

**Authors:** Lei Huang, Dongsung Kim, Xiaojing Liu, Christopher R. Myers, Jason W. Locasale

**Affiliations:** 1 Graduate Field of Computational Biology, Cornell University, Ithaca, New York, United States of America; 2 Graduate Field of Biochemistry, Molecular and Cell Biology, Cornell University, Ithaca, New York, United States of America; 3 Division of Nutritional Sciences, Cornell University, Ithaca, New York, United States of America; 4 Laboratory of Atomic and Solid State Physics, and Institute of Biotechnology, Cornell University, Ithaca, New York, United States of America; 5 Field of Computational Biology and Medicine, Cornell University, Ithaca, New York, United States of America; Institute of Agrobiotechnology, Greece

## Abstract

Fluxes are the central trait of metabolism and Kinetic Flux Profiling (KFP) is an effective method of measuring them. To generalize its applicability, we present an extension of the method that estimates the relative changes of fluxes using only relative quantitation of ^13^C-labeled metabolites. Such features are directly tailored to the more common experiment that performs only relative quantitation and compares fluxes between two conditions. We call our extension rKFP. Moreover, we examine the effects of common missing data and common modeling assumptions on (r)KFP, and provide practical suggestions. We also investigate the selection of measuring times for (r)KFP and provide a simple recipe. We then apply rKFP to ^13^C-labeled glucose time series data collected from cells under normal and glucose-deprived conditions, estimating the relative flux changes of glycolysis and its branching pathways. We identify an adaptive response in which *de novo* serine biosynthesis is compromised to maintain the glycolytic flux backbone. Together, these results greatly expand the capabilities of KFP and are suitable for broad biological applications.

## Author Summary

Metabolism underlies all biological processes, and its quantitative study is crucial for our understanding. The central trait of metabolism, metabolic fluxes, cannot be directly measured and are estimated usually through modeling. Existing modeling methods, however, are limited by poorly-characterized parameters, crude precision, or labor-intensiveness. Motivated by these limitations, and recognizing a most common goal in the field of comparing the fluxes between two conditions, we develop an extension of an existing method that takes in time-series relative-quantitation data of isotope-labeled metabolites (a kind of data that modern metabolomic technologies readily generate), and outputs the relative changes of fluxes in the metabolic networks of interest. We also carefully examine some issues on model construction and experimental design, and improve the reliability and strength of the method. We apply our method to data collected from cells in normal and glucose-deprived conditions, demonstrate the efficacy of the method and arrive at new biological insight.

This is a *PLOS Computational Biology* Methods article.

## Introduction

In recent years there has been renewed interest in metabolism resulting from discoveries of its connections to gene regulation [1], epigenetics [2], immunity [3], and pathogenesis of diseases such as cancer [4]–[6]. Independently, technological advances in metabolomics promise great improvement of our capabilities in metabolism studies and drug development [7]–[10]. However, despite the surge of interest and technological advances, quantitative systems-level characterization of the central trait of metabolism, metabolic flux, has been scarce and challenging. This is in part due to the mathematical nature of flux: rather than the amount of something that is experimentally measurable, it is defined as the rate of change in that amount and has to be inferred through modeling. Several modeling frameworks exist for the purpose. First, the century-old enzyme kinetics [11] and its systems analog, kinetic models of metabolic networks, offer a natural bridge from amount to flux, but unfortunately suffer from the “parameter problem” [12], [13] of depending on many and usually poorly-characterized kinetic parameters. Second, structural models such as Flux Balance Analysis ambitiously aim to predict global distributions of fluxes with minimal data, but the prediction accuracy is still at a stage where validation against more direct estimation results is necessary. Third, isotope-based methods exploit the elegant and powerful experimental design of isotopes, and are the workhorse for reliable flux estimations.

Among the isotope-based methods, Kinetic Flux Profiling (KFP) [14], [15] has been proven to be powerful [16]–[19], with a good balance between experimental ease, model simplicity, and prediction accuracy. In many ways complementary to Metabolic Flux Analysis (MFA) [20], another major isotope-based method which typically uses stationary isotopomer distribution data and is good at estimating relative flux distributions at branch points, KFP uses kinetic isotopomer distribution data and is good at estimating absolute flux scales along linear pathways.

**Figure 1:**
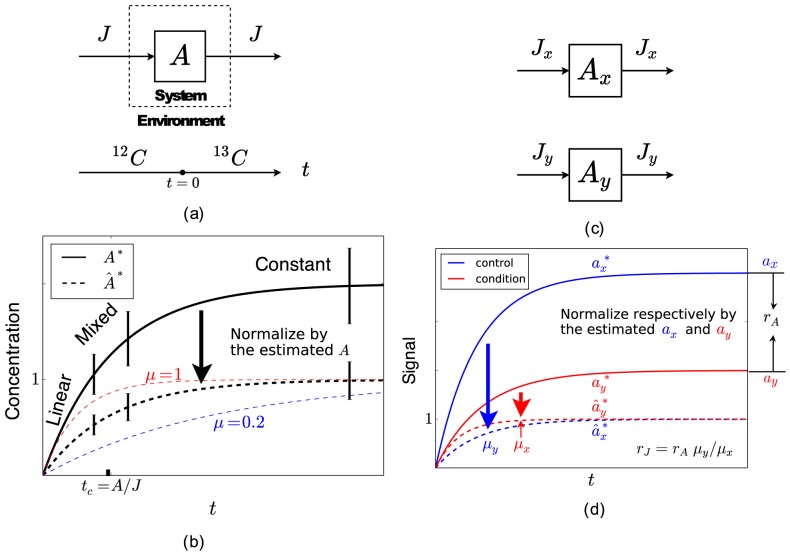
Understanding KFP and rKFP. (a) A schematic diagram of KFP applied to a toy metabolic network. At 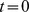, the system is switched from a 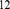
C-labeled environment to 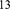
C-labeled one, and 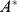 is measured at a few time points thereafter. (b) For a given trajectory of 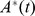 (the black solid curve), the three time regimes (linear, mixed and constant) are marked and three measurements are made (two in the mixed regime and one in the constant). Normalizing it gives 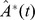 between 0 and 1 (the black dashed curve), parameterized by a single parameter 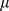, which can be estimated by comparing the normalized measurements to 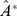
's of different 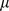
's (the red and blue dashed curves). (c) A schematic diagram of rKFP applied to the same network in (a). Relative quantitation is performed on 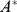 in two conditions (with subscripts 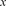 and 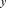 respectively) with the goal of estimating 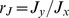. (d) The ratio in 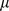 between 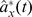 and 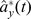 is 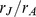 (Eq. 6), and since 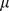
's and 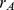 are identifiable from relative quantitation, so is 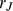.

The basic idea of KFP can be illustrated using a toy metabolic network. Consider a system of only one metabolite 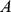 connected to the environment by an influx 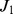 and an outflux 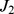; the system is at steady state so 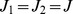 (Figure 1a). KFP works by switching the system from a 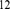
C-labeled environment to a 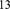
C-labeled one at time 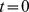, measuring the concentrations of 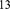
C-labeled 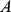 (termed 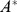) at several time points thereafter, and estimating 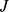 from the time series data of 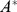. After the switching of environment, 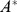 will gradually infiltrate the pool of 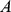 as a result of 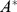-carrying influx, with the dynamics described by
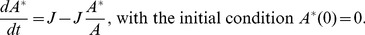
(1)


The two terms 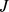 and 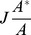 in the right-hand side respectively describe the infiltration of 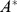 into the 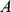 pool by the influx and the opposing depletion by the outflux, and their net difference describes the rate of change of 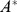. The equation is a first-order linear ordinary differential equation (ODE), and can be solved using standard techniques (see Text S1). Its solution is the simple exponential approach function
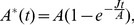
(2)


and geometrically corresponds to a family of curves parameterized by 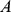 and 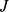 (Figure 1b).

With some measurements of 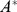 along the curve, parameters 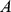 and 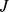 can be estimated in a standard way: a least-squares fitting algorithm gives the best fit, and sensitivity analysis or Monte Carlo simulations give the uncertainties. However, it helps to understand *why* KFP should work in this case. First, it is easy to see from Eq. 2 that parameter 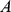 determines the saturation level of 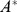 and 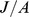 determines the rate at which the saturation level is approached; in other words, 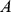 determines the *scale* and 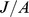 determines the *rate*. To highlight this, we define a rate parameter, 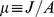; its inverse, 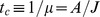, is conventionally called the characteristic time-scale and numerically corresponds to the time needed to go from the initial condition to 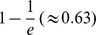 of the saturation level. Second, like the familiar Michaelis-Menten hyperbolic curves, the exponential approach curves can also be thought as having three regimes, defined with respect to the characteristic time-scale: the linear regime when 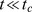, the constant regime when 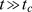, and the mixed regime in between when 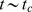 (Figure 1b). Measurements of 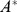 in the linear regime are informative of only 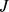 (for the slope of that regime is 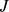), the constant regime of only 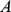 (for 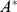 in that regime is constantly 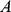), and the mixed regime of both. In practice, however, usually only the constant and mixed regimes are measured due to their experimental accessibility. Finally, after 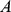 is estimated from measurements in the constant regime, the estimation of 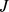 from measurements in the mixed regime can be understood in the following way: normalize all measurements by the estimated 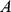 to describe the normalized variable 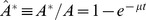; parameterized only by 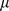 and now increasing from 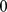 to 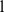, 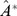 changes from a sharply rising curve to a gently rising one as 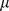 decreases; the normalized measurements in the mixed regime nail down the specific 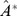 within the family of curves, together with 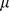 and 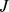 (Figure 1b). The discussion above can be succinctly summarized as “parameters 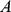 and 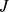 are *identifiable* when applying KFP to the system in Figure 1a”. Later in the paper we will see how the reasoning described here in terms of normalization and rate can be used again to understand the estimation of relative changes of fluxes and the ratios of pool sizes, and the selection of measuring times.

Applying KFP to systems of arbitrary size and network topology and with multiple influxes is less straightforward and requires care [17]. When a reaction involves more than one substrate, the labeling states are no longer just labeled or unlabeled as in KFP, and tracing the origins and fates of 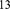
C labels requires the knowledge of Carbon Transition Map [21] of the reactions. The assumption of irreversibility can eliminate this complication for decomposition reactions, but is only valid for far-from-equilibrium ones. Also, multiple influxes to a system complicate modeling the dynamics of 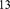
C-labeled metabolites in the same way as multi-substrate reactions do. For this reason, KFP works best for systems consisting of mono-molecular reactions, and works for general systems only through gathering additional information, making assumptions, or using only part of the data that is more amenable to model. The last strategy corresponds to the idea used in an extension of KFP called extended KFP (or eKFP) [17], and is relevant in our later discussion on the capacity of KFP and our extension of it in studying metabolic cycles.

Two considerations motivate us to extend KFP beyond its current scope. First, KFP requires *absolute quantitation* of metabolites, meaning that their absolute concentrations have to be measured, while many experimental techniques such as mass spectrometry can only perform *relative quantitation* readily, meaning that the measurement output is scaled from the absolute concentration by a metabolite-specific unknown constant; going from relative quantitation to absolute quantitation typically requires performing relative quantitation on some reference samples whose absolute concentrations are known, which can often be a challenge due to the increased effort of both additional experiments and procurement of reference samples. Second, often it is the relative changes of fluxes (or biological quantities in general) between two conditions that are of interest or biological relevance (*e.g.*, wildtype *vs.* mutant, control *vs.* drug-treated; [16], [22]), and estimating the absolute fluxes of the two conditions only to get their ratios is inefficient (the information regarding their scales is eventually discarded) and roundabout (three rounds of estimation are carried out, one for each condition and one for their ratio).

In this paper, we report an extension of KFP that can estimate the relative changes of fluxes using only relative quantitation, which we call rKFP, hence addressing the two considerations above. To improve the reliability and strength of KFP and rKFP, we examine some issues in the application of the methods, on both setting up models and selecting measuring times. Finally, we apply rKFP to experimental data collected in normal and glucose-deprived conditions, estimating the relative flux changes in glycolysis and its branching pathways and arriving at new biological insight.

## Results/Discussion

### Extending KFP to Estimate Relative Flux Changes

Consider again the toy system in Figure 1a, now in two different conditions; the same experimental procedures of switching environment at 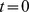 and subsequent measurements of 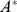 apply, but only with relative quantitation (Figures 1c and 1d). The aim is to estimate the relative change of 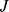 between the two conditions.

We start by writing down the relationship between the relative quantitation measurements (which we call signals) and the absolute quantitation measurements (concentrations) for the two conditions:
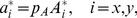
(3)


where an upper-case letter denotes concentration and lower-case signal, 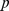 their ratio, superscript 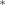 a labeled quantity, and subscripts 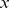 and 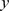 quantities for the two conditions respectively (a list of notation can be found in Table 1). Since now we can only perform relative quantitation and hence 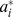 becomes the measurable, we establish its dynamics by plugging in the dynamics of 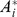 which we have solved in studying KFP:
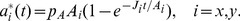
(4)


**Table 1:** Definitions of variables and their identifiabilities in rKFP.

Symbol	Meaning	Identifiable in rKFP
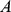 (uppercase)	metabolite 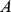 or it pool size (absolute quantitation) 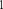	No
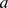 (lowercase)	signal of the pool size (relative quantitation)	Yes
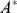	concentration of 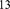 C-labeled 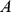 (absolute quantitation)	No
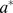	signal of 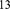 C-labeled 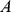 (relative quantitation)	Yes
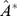	the fraction of 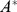 in 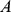 , 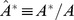	Yes
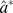	the fraction of 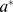 in 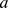 , 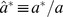	Yes
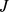	flux	No
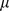	rate, 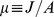	Yes
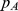	proportionality constant between signal (relative quantitation) and concentration (absolute quantitation) of 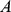 , 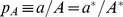	No
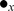 (subscript 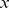 )	a quantity of control	–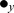 (subscript 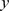 )	a quantity of condition	–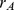	relative change of pool size, 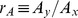	Yes
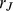	relative change of flux, 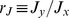	**Yes**
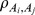	ratio of pool size, 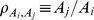	**Yes**

1: Which one of the two possible meanings is meant should be inferable from the context when not specified; it is a slight abuse of notation customary in the field (*e.g.*, [46]).

The above two equations highlight a simple but important fact: 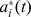 is simply 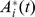 scaled by an unknown constant 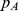, and they share the same intrinsic dynamics. Therefore, the reasoning of normalization and rate described in the discussions of KFP in the introduction appears even *more* natural in this situation: although relative quantitation leaves us oblivious to the scale, we can still normalize the measurements to uncover the rate.
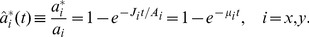
(5)


Defining the relative changes of pool size 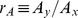 and flux 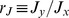, the relative change of 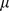 can then be expressed in terms of 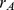 and 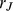:
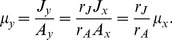
(6)


Since 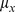 and 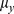 are identifiable using normalized measurements in the same way as 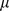 described in the introduction, and 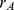 is obviously identifiable (Figure 1d), so is 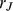. This concludes our explanation of why rKFP should work for the toy system in Figure 1c.

To summarize the mechanics of rKFP for the system, one sets up the following two equations:
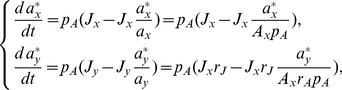
(7)


which form a model that is parameterized by 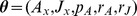, and predicts 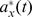 and 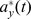 (the solutions are Eq. 4); measurements of 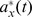 and 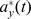 allow for estimating 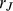 (and 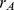) with precision (identifiable). Two remarks follow: first, while parameters 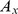, 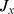 and 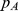 are obviously non-identifiable, two of their functions, 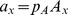 and 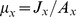, are, amounting to two constraints on 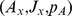; second, in light of the constraints, the model can be parameterized in other natural ways: for example, one can replace 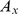 with 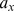, or 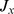 with 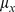, and the resulting new parameterization would be as interpretable.

For larger networks consisting of single-substrate reactions arranged in linear pathways and branch points, rKFP proceeds in a similar way: equations like Eq. 7 are constructed for each metabolite in the network, and relative quantitation measurements of the metabolites allow for estimating the relative changes of all the independent fluxes with precision. However, for metabolic networks involving reactions of multiple substrates, especially cycles (multi-substrate reactions are usually present in cycles as part of the network design; see supplementary Text S1), rKFP in its above form cannot handle the situation. Fortunately, a variant of KFP, termed extended KFP (or eKFP), has been developed to overcome the problem [17], and its rKFP version, which we term reKFP, can estimate the relative flux changes for cycles without having to deal with the complications of modeling carbon transitions that arise in multi-substrate reactions [23]. The essential idea of eKFP is that while there can be multiple labeled states for the reactants in multi-substrate reactions and keeping track of their transitions can be complicated, there is always only one unlabeled state and modeling its dynamics is relatively simple and stays within the KFP framework; the downsides are that only a fraction of the information in the data is used and that the measurements of the unlabeled metabolites have to be accurate which sometimes can be nontrivial to achieve due to media contamination. A detailed discussion of how reKFP can be applied to cycles is contained in supplementary Text S1.

Before we conclude the discussion of rKFP, we mention one more nontrivial quantity identifiable from relative quantitation, the ratio of pool sizes. As an illustrating example, consider a metabolic pathway of two metabolites with relative quantitation; we again normalize the measurements to uncover the intrinsic dynamics. The idea is that the further the second metabolite lags behind the first one, the more abundant it is compared to the first one (Figure S1a).

Formally, one can plug 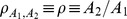 and 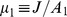 into the normalized 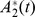 (Text S1 contains a derivation of 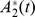):
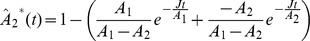


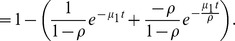



Since 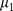 is identifiable from relative quantitation of 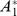, the single parameter that is left, 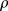, determines how much 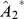 lags behind 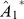 and is identifiable from the comparison (Figure S1b). In the introduction we discussed the general challenge of absolute quantitation, but here we show that if absolute quantitation can be performed on, or good prior knowledge exists for, *some* metabolites (*e.g.*, the cellular glucose concentration is known to be about 5 mM [24]), this information of scale can be propagated across the network to *other* metabolites through the estimates of pool size ratios, estimating absolute concentrations from relative quantitation.

### Missing Data: Effects and Pitfalls

Despite the great advances in metabolomic technologies, it is nevertheless common to have missing data. It is a result of the chemical properties of metabolites: some are too unstable to be accurately measured, and some isomers are too similar to each other to be distinguishable. To set up a computational model for KFP or rKFP under missing data, one in principle has the following options: (1) use a reduced model where the network components corresponding to the missing data are removed; (2) use a full model where all components are kept and the part of the model corresponding to missing data represents additional degrees of freedom unconstrained by data; (3) use the full model but incorporate prior information for the part of model uncovered by data; (4) spend additional effort to collect all data and use the full model. We observe that a common practice in applying KFP is to choose the first option and use reduced models when there is missing data (*e.g.*, [16]). We acknowledge that this option is tempting and reduced models do offer many advantages such as conceptual simplicity and computational manageability (after all, “all models are wrong; some are useful”); however, because of the potential bias reduced models might introduce to parameter estimation and model prediction, we believe that their use would be better justified after a careful consideration of their effects.

**Figure 2:**
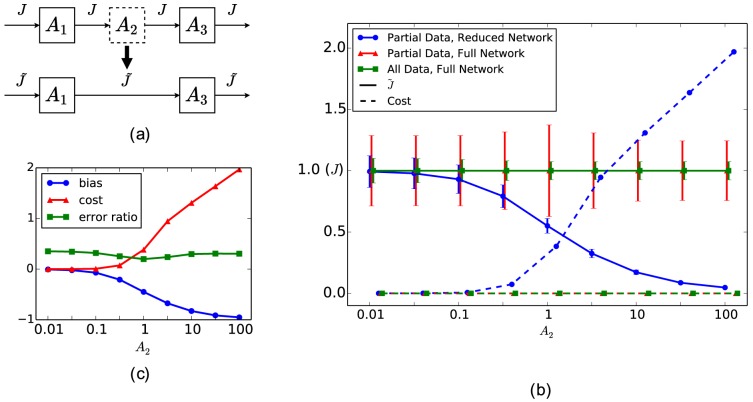
Metabolite removal in KFP. (a) A schematic diagram of getting the reduced model through metabolite removal in KFP. Dashed squares represent metabolites removed in the reduced model; thick dark arrow represents reduction; 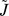 represents the estimated 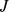 (potentially biased). (b) The estimation results for the three options. The solid curves represent 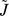, the dashed curves represent the cost of fitting (normalized by the number of data points to be comparable across options), and three colors represent the three options. Parameter values used for generating the simulated data: 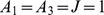 (overall patterns independent of the choice here). (c) The estimation results in (b) in terms of the three summary statistics.

Consider the following example. Figure 2a provides a cartoon of a typical situation in applying KFP: in a linear pathway of three metabolites, 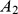 is hard to measure and therefore constitutes missing data. The above four options concretize to the followings: (1) use the reduced model consisting of only 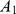 and 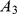; (2) use the full model consisting of all three metabolites with 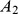 uncovered by data; (3) use the full model but put a prior distribution (in the Bayesian sense) on the 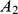 pool size based on previous knowledge; (4) try to collect 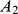 to complete the data and use the full model. We note that the desirability of option (3) depends on what prior distribution is available and how close it is to the true value, *i.e.*, how well one *a priori* knows the missing piece. Hence it has to be judged on a case-by-case basis and cannot be discussed generally. We therefore exclude option (3) in our subsequent analysis, and only note that in the limit of a correct tight prior the case converges to option (4) of completing the data, and in the limit of a loose prior the case converges to option (2) of effectively having no prior information.

We call this scenario of missing data in Figure 2a *missing metabolite*, and the corresponding procedure of constructing reduced models *metabolite removal*. We identify and name three additional common scenarios of missing data with their associated reduction procedures: first, *missing pathway*, where a branching pathway has poor data coverage with most metabolites unmeasured, associated with *pathway removal*, where the branching pathway is removed from the model; second, *undistinguished metabolites*, where the individual identity of a set of metabolites in a measurement cannot be resolved, associated with *lumping*, where the undistinguished metabolites are lumped into a single pool; third, *unknown reversibility*, where the extent of reversibility of a reaction is unknown, associated with *assuming irreversibility*, where potentially reversible reactions are modeled as irreversible for simplicity. In the remainder of this section, we describe in details the effects of metabolite removal on both KFP and rKFP, for this case is the simplest and hence most illustrative, and also for this case turns out to be important (see below); after that we briefly describe the results on pathway removal and lumping, leaving the details to the supplementary text; results on assuming irreversibility are discussed in the next section.

Back to Figure 2a, intuitively, using the reduced model with 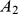 removed would *underestimate* 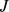, for two reasons. First, since the influx 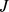 is 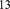
C-saturated and constant with time, after time 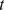 one would expect 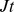 amount of 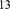
C in the system, distributed across different metabolite pools; removing 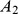 from the network excludes the 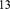
C in that pool, causing an underestimation of the 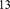
C in the system and hence an underestimated 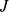. Second, the presence of any metabolite pool slows down the infiltration process of 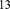
C along the network, and if the slow-down of the infiltration by the 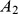 pool is concealed by removing 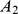 from the network, the slowed infiltration would be attributed to a lower 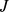. Both factors become more pronounced as the pool size of 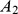 increases, and so should be the underestimation.

Figure 2b shows the results for the three options. First, using the reduced model indeed underestimates 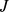, and it worsens as 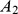 increases (the blue solid curve), confirming our intuition. Second, using the reduced model decreases the goodness-of-fit between the model and the data, quantified by the cost of fitting, and it increases with 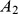 as well (the blue dashed curve). Third, using full models causes no bias or cost (red/green and solid/dashed curves). Fourth, the extra hard work of collecting the data of 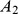 pays off by shrinking the uncertainties of the estimated 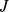 (the red *vs.* the green error bars). These observations suggest the following three summary statistics:

- bias, defined as 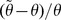, where 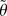 and 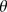 are respectively the estimated and true value of a parameter (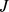 in this case).
- cost, the cost of fitting using the reduced model.
- error ratio, defined as 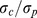 where 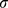 is the standard error of the parameter estimate and subscripts 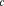 and 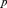 refer respectively to the cases of complete and partial data.

These statistics summarize the effects and pitfalls of missing data: error ratio quantifies their adverse effects on parameter estimation (or, looking optimistically, the reward of completing them), and bias and cost quantify the harm of using reduced models. Figure 2c re-plots the results in Figure 2b in terms of the three summary statistics. From these plots we can see that KFP is rather sensitive to metabolite removal: in the case of the toy model, a missing metabolite of pool size equal to others causes roughly a 50% bias. The same should hold for general pathways: if the total pool size of the missing metabolites in a pathway is relatively large, the bias should be considerable. For this reason, we give the general suggestion that, unless the missing metabolites are known *a priori* to have small pool sizes, one should use full models in KFP.

**Figure 3:**
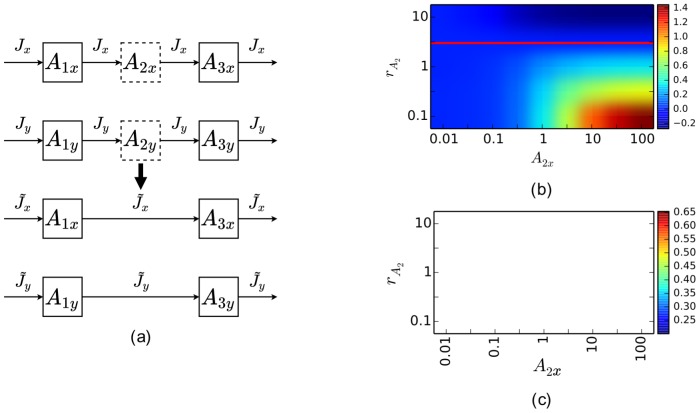
Metabolite removal in rKFP. (a) A schematic diagram of getting the reduced model through metabolite removal in rKFP. The same figure scheme as in Figure 2a applies here. Parameter values used in generating the simulated data: 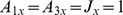, 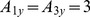, and 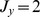. (b) Dependence of bias on the pool size of the missing metabolite in two conditions. (c) Dependence of error ratio on the pool size of the missing metabolite in two conditions.

We next examine how metabolite removal affects rKFP (Figure 3a). We have concluded that removing 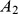 in KFP underestimates 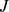 and the underestimation worsens as 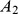 increases. Since 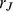 is the ratio between 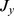 and 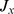, we expect that the bias in 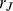 depends on 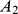 in the two conditions, 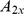 and 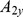: if 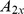 is large and 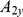 small, 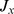 is implicitly more underestimated than 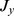 (implicitly as rKFP does not explicitly estimate 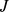), giving an overestimated 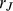, and in the same way a small 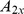 and a large 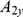 give an underestimated 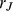. Like KFP, we expect the bias to increase with the pool size difference of 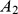 between two conditions.

Figure 3b plots the bias of 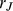, and confirms our intuition. However, an important feature distinguishes the case in rKFP from KFP. Figure 3b shows a red line where the relative change of 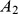 is the same as 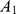 and 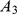, and above the red line 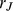 is underestimated and below overestimated; in other words, when the relative change of 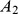 pool size exactly matches the others in the pathway, 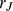 is estimated without bias. This hints at another important advantage of rKFP over KFP: bias can be introduced to the two conditions in such a similar way that some of it is canceled out. The question remains of how likely the pool size of a metabolite changes in a similar way to all others in a pathway. Figure S3 plots the fitting of experimental data of glycolysis in two conditions, and shows that the relative changes in pool size of all metabolites in the pathway fall within a factor of five. From an enzyme kinetic perspective, this says that the reactions along the pathway are in similar *elasticity* regimes [25], which is also consistent with the consensus that reactions in a pathway in general share the *flux control* [26]. We hence believe that in rKFP some of the bias is indeed canceled out which makes it *more robust* to metabolite removal than KFP. However, we also note in Figure 3b that the bias worsens quickly as the relative change in pool size of different metabolites diverge: when it is off by a factor of three, say, 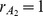 (while 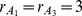), the bias becomes about 40%. This implies that in a typical situation despite the canceling the remaining bias is still too large to justify using the reduced model, and hence we again suggest using full models in rKFP for missing metabolites. The ideas of canceling and metabolites along a pathway changing pool sizes similarly, however, find their use in the next section where they serve to justify using reduced models for unknown reversibility.

To summarize, we have demonstrated above the effects of missing metabolites on (r)KFP and the pitfalls of removing them; we suggest that unless one has good prior knowledge about the missing pool size or its relative change between two conditions that ensures a small bias, full models should be used. The approach we use for the demonstration is an intuitive and computational one, and in supplementary Text S1 we outline a complementary and analytical method that allows for a more precise description and deeper insight into the dependence of bias on relevant parameters. Also included there are some detailed studies of how two other reduction scenarios, pathway removal and lumping, might affect KFP and rKFP. We summarize the results in the following: first, both KFP and rKFP are rather robust to the removal of a minor branching pathway (that is, a branching pathway that does not carry the majority of the flux), while using the full model greatly inflates the confidence interval of the parameter estimates due to the additional degrees of freedom; second, since lumping is usually applied to isomers which are often close to chemical equilibrium, it is generally rather innocuous in light of the results in the next section. In a later section on data analysis, we see that the results in this section are verified by comparing the estimation results of different models.

### Modeling Reversible Reactions

Biochemical reactions often operate close to equilibrium [27] (for example, it is conventionally thought that this applies to seven out of the ten reactions in glycolysis [28]), and a reaction's distance to equilibrium is commonly quantified by the difference in Gibbs free energy, 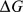, between substrates and products: the closer 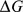 is to zero, the closer the reaction is to equilibrium. One of the properties of a close-to-equilibrium reaction is that both of its forward and backward fluxes are large and most of them are canceled out to give a much smaller net flux; quantitatively this is described by the *flux-force relationship*: 
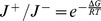
, where 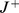 and 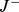 are the forward and backward flux respectively, 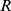 the gas constant and 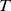 the temperature [29]. Quantity 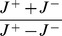, the total flux over the net flux, therefore describes how much “futile” work the reaction does compared to “useful” work, and predicts from the flux-force relationship that as the reaction approaches equilibrium, exponentially more fluxes simply go back and forth compared to the net flux (Figure S2a). This has an important implication in the context of (r)KFP: when the reversible fluxes are large compared to the net flux, the time-scale of the mixing of substrate and product pools is much shorter than that of the infiltration of the 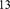
C-labeled metabolites in each pool, making the two pools effectively one as far as (r)KFP is concerned (Figure S2b).

**Figure 4:**
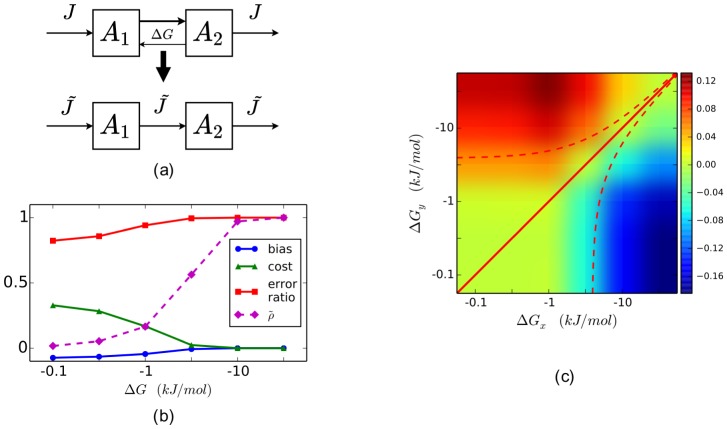
Modeling reversible reactions in KFP and rKFP. (a) A schematic diagram of the toy system considered here; for rKFP two copies of each network are similarly made as in Figure 3a. Parameter values used in generating the simulated data: 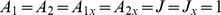, 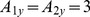, and 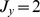. (b) Dependence of the summary statistics on 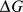 in KFP. (c) Dependence of the bias of 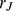 on 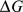 of the two conditions in rKFP. The red solid diagonal line corresponds to 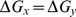 where there is no bias. The red dashed curves correspond to a five-fold difference in the relative pool size changes between the substrate and product, a range we observe in our data.

This suggests another potential issue in setting up models for KFP or rKFP: ignoring the reversible fluxes when the reaction is close to equilibrium, as is commonly done, might introduce bias. To check this, we again use a toy system (Figure 4a) and, similar to the case of missing metabolite, conceive three options for modeling a reversible reaction: (a) model it as irreversible; (b) model it as reversible and let 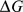 be a free parameter; (c) model it as reversible and measure 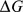 to some precision (which typically requires absolute quantitation). Figure 4b plots the summary statistics for KFP, which shows that there is a small bias and a moderate cost when a highly reversible reaction is assumed irreversible. It suggests that KFP might be robust to assuming irreversibility; however, even the small bias can be avoided since in KFP 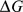 can be calculated and is not missing information: KFP uses concentration data, which can be used to calculate 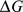 through its definition (see below); therefore one can explicitly incorporate in the model 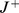 and 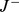 which depend on the parameter 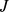 through the relationships 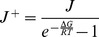 and 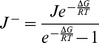. Also plotted is the estimated pool size ratio between 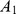 and 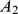 with one being the true value (the purple dashed line), which shows a vast underestimation when the reaction is close to equilibrium; this can be explained by the following: as the reaction becomes more reversible, 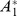 and 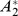 share the dynamics to a greater extent (Figure S2b), and 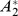 closely following behind 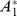 is interpreted by the algorithm as resulting from 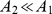 (Figure S1a).

Figure 4c illustrates how ignoring reaction reversibilities biases rKFP, which can be understood in a similar way as Figure 3b in using reduced models. First, the direction of the bias depends on 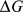
's of the two conditions; since ignoring reaction reversibilities underestimates 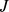 in KFP (Figure 4b), if the reaction is more reversible in control (x) than in condition (y), then 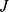 would be more underestimated in control, giving an overestimated 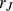 (the red region), and *vice versa*. Second, if 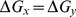, then 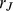 is estimated without bias (the red line).

We naturally wonder how likely the change of 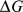 falls around the red line, and find that its definition offers the clue. Since 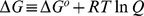, where 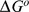 is the standard Gibbs free energy change and 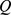 the reaction quotient defined as the product concentrations divided by the substrate concentrations. For a mono-molecular reaction with 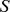 the substrate and 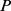 the product, 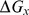 and 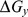 can be related in the following way:
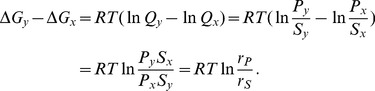
(8)


In other words, the change in 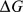 depends on the relative changes in pool size of 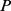 and 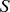. In the previous section we conclude that the relative changes in pool size of the metabolites along a pathway are likely to be similar, and Figure 4c shows that, *unlike* the case of metabolite removal, the bias increases *slowly* as the relative pool size changes of different metabolites diverge (the red dashed curves correspond to a five-fold difference in the relative pool size changes, a range of variation we observe in our glucose-deprivation data, and delineate a region surrounding the diagonal line of very small bias). We therefore conclude rKFP should be robust to assuming irreversibility and reduced models can be used.

We last note that, like the case of pathway removal discussed in the supplementary text, using the full model in this case and incorporating 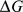
's as free parameters would leave the model underconstrained by data and lead to huge uncertainties in parameter estimates, since one additional parameter would be accumulated for each reaction modeled as reversible. Incorporating prior information on 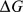
's would help, but at a systems level the information is scarce as it requires accurate absolute quantitation [27] and predictive computational methods are still being developed [30]. It is also our experience that rKFP models constructed this way with additional 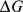 parameters are prone to numerical problems in data fitting and parameter sampling, since for each reaction modeled as reversible two copies of 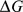 (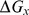 and 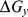) need to be made and they are entangled with parameters of relative pool size changes through Eq. 8. In light of all these challenges, the robustness of rKFP to assuming irreversibility is advantageous.

### Selecting Measuring Times

In addition to the issues on setting up models as discussed in the previous two sections, we have also investigated the experimental design issue of selecting measuring times for a metabolic pathway in (r)KFP. We list below the main conclusions, and leave the derivation details to the supplementary text.

First, we note that the problem can be formulated as a classical experimental design one [31]: select a set of measuring times such that the errors of the estimated parameters (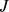
's in KFP and 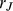
's in rKFP) are the smallest. However, such an approach suffers from computational intractability, physical uninterpretability and unrealisticness (Text S1).

We choose to adopt an alternative approach for which there is a physical intuition: since the dynamics of the metabolites in a pathway are linear combinations of exponential functions (Text S1), and in the introduction we show that for a single exponential function, there is a well-defined characteristic time-scale, we wonder if we can make use of this special mathematical structure and target the measuring times on the different time-scales.

We show that for the first metabolite in a pathway, if a late time has been measured so that its (relative) scale is estimated, the optimal measuring time would be around 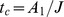, the characteristic time-scale. This highlights another significance of 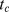: if the curve of 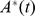 in Figure 1b is held fixed at two ends and the middle left wiggling as 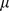 changes, placing a pin at the characteristic time-scale leaves the least wiggle room.

We also show by using a similar reasoning that for the second metabolite in a pathway the optimal measuring time is around 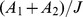, and similarly for the k-th metabolite around 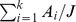. That is, the measuring time for the k-th metabolite should be around the sum of the characteristic time-scales of all the metabolites up to the k-th one; this makes intuitive sense, given that the dynamics of 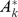 is a mixture of k time-scales due to the actions of the k pools. We therefore provide the following practical suggestion: for a metabolic pathway of n metabolites with a rough prior guess of the flux (and pool sizes for rKFP), after measuring at a late time so that the (relative) scales of all metabolites can be estimated, other measurements should go to 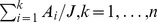. Given this scheme of selecting measuring times, it is calculated through simulation and shown in the supplementary text how the precision of estimated parameters depends on the number of data points and how those data points should be optimally distributed across different metabolites and times.

We last note that a typical metabolic network exhibits strong separation of concentration scales. For the example of glycolysis, one source reports that the most abundant metabolite (glucose) is about 35 times more than the second most abundant one and the full range of variation spans over three orders of magnitude [28]. This has an important implication: the most abundant metabolite dominates the numerator of 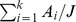, and hence the sampling time. If the dominant metabolite happens to be the first one, as is suggested for glycolysis in [28], then the whole pathway effectively shares one dynamics and measuring time.

### Applying rKFP to Glucose Deprivation Data

In this last section, we apply rKFP to experimental data collected on glycolysis and its two main branching pathways (pentose-phosphate pathway, or PPP, and serine synthesis pathway) from cells in normal (5 mM glucose) and glucose-deprived (0.5 mM glucose) media conditions, and estimate the relative changes in the fluxes of the three pathways.

**Figure 5:**
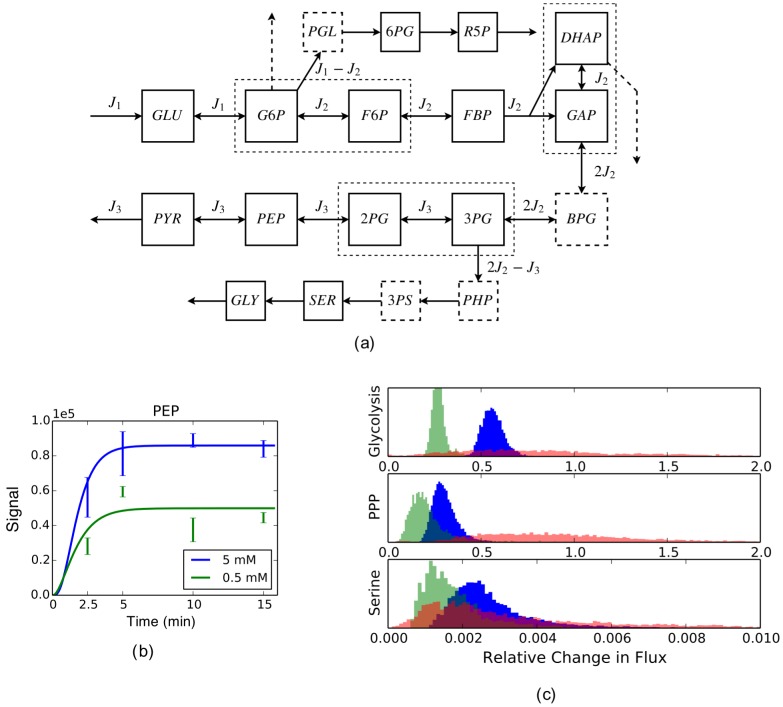
Analysis of experimental data. (a) The diagram of glycolysis and its two branching pathways used in the analysis, where dashed squares (*e.g.*, BPG) represent missing metabolites, dashed arrows represent missing pathways, and dashed rectangles containing solid squares represent undistinguished metabolites. Abbreviations for metabolites: GLU: glucose; G6P: glucose-6-phosphate; F6P: fructose-6-phosphate; FBP: fructose-1,6-bisphosphate; DHAP: dihydroxyacetone phosphate; GAP: glyceraldehyde-3-phosphate; BPG: 1,3-biphosphglycerate; 3PG: 3-phosphoglycerate; 2PG: 2-phosphoglycerate; PEP: phosphoenolpyruvate; PYR: pyruvate; PGL: 6-phosphogluconolactone; 6PG: phosphogluconate; R5P: the pool of ribose 5-phosphate, ribulose 5-phosphate and xylulose 5-phosphate; PHP: phosphohydroxypyruvate; 3PS: 3-phosphoserine; SER: serine; GLY: glycine. (b) An exemplary plot of the data of a metabolite and its fit. Plots of all metabolites and their fits can be found in Figure S3. (c) Histograms of 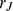
's as generated by sampling the corresponding posterior distributions in a way detailed in the Methods. Glycolysis flux refers to 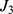 in the diagram, PPP flux 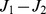, and serine synthesis flux 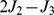. The three histograms for each flux correspond to three different modeling choices described in the text.

Figure 5a shows the diagram of the network, where the upper pathway branching off glycolysis at G6P is (part of) the PPP, and the lower pathway branching off glycolysis at 3PG is (part of) the serine synthesis pathway; two additional branching pathways, glycogen synthesis pathway and glycerol synthesis pathway, emanating from G6P and DHAP, respectively, are also portrayed (the dashed arrows). Further described in the network diagram is our data coverage, which contains all types of missing data we have considered: data of metabolites BPG and a few others in branching pathways are missing, data of the glycogen and glycerol synthesis pathways are missing, isomers such as G6P/F6P, GAP/DHAP and 3PG/2PG are not distinguished, and the extent of reversibility of the reactions is not known.

We set up three computational models for rKFP, corresponding to three different choices of treating missing data as discussed in earlier sections, and the estimation results of all three models are shown in Figure 5c: dark blue histograms correspond to the choice recommended in the earlier sections, namely, using the full model only for missing metabolite, light green the choice of using the reduced model for all types of missing data, and light red the choice of using the full model for both missing metabolite and pathway. We make two observations from the comparison of the histograms: the green histograms are tighter than the blue but shifted, and the red histograms have similar averages to the blue (except for PPP) but much larger variances.

The observations support our recommended choice. The shifted green histograms relative to the blue suggest that metabolite removal introduces significant bias, likely because the pool sizes of the missing metabolites change differently from the other metabolites. The flattened red histograms relative to the blue suggest that keeping in the model the missing pathways with no data leaves the estimation greatly underconstrained; on the other hand, their similar averages suggest that the bias introduced by pathway removal is small in this case, likely because the missing pathways have much lower fluxes than the main glycolysis pathway (see the supplementary text) which is consistent with some previous studies (*e.g.*, [16], [32]; also see Figure S3) and that the cell line in our study exhibits the Warburg effect [4] with most of the glycolytic flux going to lactate fermentation. In summary, through the comparison of different models for both toy and real networks, we have chosen the following choice: in the face of missing data, we keep the model components that matter (metabolites) and leave out those that do not and would make the estimation underconstrained or numerically unstable (pathways, distinct pools and reversibilities). For future applications of rKFP, we suggest users either follow our recommended choice, or, better yet, set up different models and compare the results to verify the choice as is done here.

From the parameter distributions under our recommended model choice, we observe that, despite a 10-fold decrease in the media glucose concentration, glycolysis and PPP fluxes are reduced by about 40% and 60% respectively, while the serine pathway is basically incapacitated by the glucose deprivation. We make the following interpretation of the results. As it is generally believed that tumor cell proliferation sometimes requires metabolic adaptation to a microenvironment deprived of glucose, the effect of glucose deprivation on metabolism has been of interest [33]–[35]. Also of interest is the activity of serine biosynthesis pathway for its implication in tumorigenesis [36]. Here we show that when the external glucose source is depleted, the cells adapt their metabolism by largely maintaining the backbone of glycolysis flux at the expense of serine biosynthesis flux. This suggests the possibility of additional contextual requirements of PHGDH, the enzyme that diverts flux from glycolysis into *de novo* serine biosynthesis, and the possibility of other mechanisms for serine utilization in the condition of low glucose availability.

## Materials and Methods

### Computation

Individual steps of the computation in this study are listed and explained below using KFP as the example (many of them use *SloppyCell*, a Python package originally developed for analyzing biochemical networks [37]). Relevant Python codes are deposited at http://github.com/leihuang/rkfp.

- Encoding models: models are encoded in an SBML-compliant format [38] in SloppyCell and mathematically correspond to systems of ODEs of 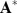, the concentration vector of labeled metabolites.
- Simulating models: *daskr*, an algebraic-differential equation solver [39] used in SloppyCell numerically integrates the models.
- Generating simulated data: given the integrated trajectories of 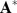, simulated data are generated by a selection of measuring times following the suggestions in the section on selecting measuring times, and the associated noise is generated by choosing a constant value when deriving analytical results for its tractability and assuming to be proportional to data when carrying out simulations for its realisticness (a proportionality constant of 0.2 is used).
- Estimating parameters: letting 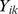 be the measurement of the model prediction 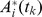 and 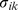 be the noise associated with 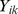, one defines the cost of fitting as a function of parameter 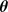: 
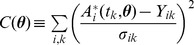
 (also known as 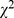); SloppyCell uses the Levenberg-Marquardt algorithm [40] to minimize 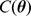 and find the parameter estimate 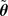. In our analysis of experimental data where the model is large and the data is noisy, we find that having a good initial guess is crucial [41] and having a large L-M parameter 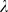 and a small trust region helps.
- Integrating sensitivity curves: SloppyCell calculates 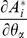 as a function of 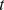 in an accurate way (more details in Text S1), which is important for calculating Jacobian and errors (below);
- Estimating errors: (1) construct the Jacobian of the model, 
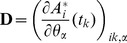
 by filling a matrix with the sensitivities at the assumed measuring times along the sensitivity curves; (2) normalize each entry by 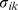; (3) perform the singular value decomposition 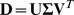; (4) the square roots of the diagonal entries of the matrix 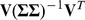 give the estimates of errors.
- Generating posterior distribution: assuming Gaussian-distributed measurement noise, the measurement of 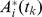 is also Gaussian distributed: 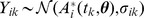; treating the parameter estimation problem in the Bayesian framework and assuming an uninformative prior, 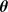 has a posterior distribution with a probability density proportional to that of observing 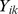 in 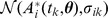, following the Bayes' rule: 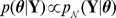; Metropolis algorithm [42] is used to sample the posterior distribution in SloppyCell, and parameters of 100,000 steps, 1% burn-in and 50-step thinning interval [43] are used for generating the distributions in Figure 5c.

Note that the last two points constitute the two standard ways of estimating parameter uncertainties: the first one also goes by the name of *sensitivity analysis* or *delta method*, and is computationally cheap but less accurate; the second one is also known as *ensemble method* [44], and is computationally expensive but more accurate. In this study, simulations intended to illustrate basic principles use the first method, and data analyses intended to draw realistic conclusions use the second.

### Experiments

Experimental procedures of cell culture, metabolite extraction, mass spectrometry, liquid chromatography and data processing follow those in [45]. On the procedures specific to rKFP, HCT116 human colon cancer cells cultured in 6 well dishes with RPMI 1640 were washed with PBS and transferred to two media with 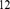
C-glucose of concentrations 5 mM and 500 µM respectively, where they were incubated for 2.5 hours before switching to media with 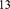
C-glucose of the same concentrations; relative quantitation of triplicates were then performed on the cells at 0, 2.5, 5, 10 and 15 minutes after the switching.

## Supporting Information

Text S1 A file containing three supplementary figures, a derivation of the solution for KFP applied to a linear metabolic pathway, a discussion on applying (r)KFP to metabolic cycles, the description of an analytical approach to studying the effects of removing metabolites and pathways, and effects of pathway removal in KFP and rKFP, and some detailed results on the effects of missing data and selecting measuring times.
(PDF)

Dataset S1 ^13^ *C*-labeled relative-quantitation data collected from cells in normal and glucose-deprived media used for the analysis.
(CSV)
