# Supplementary material for: Estimating Relative Changes of Metabolic Fluxes
Source: PLoS Comput Biol. 2014 Nov 20;10(11):e1003958. doi: 10.1371/journal.pcbi.1003958 (PMC4238958; doi:10.1371/journal.pcbi.1003958)
Supplement: Text S1 — A file containing three supplementary figures, a derivation of the solution for KFP applied to a linear metabolic pathway, a discussion on applying (r)KFP to metabolic cycles, the description of an analytical approach to studying the effects of removing metabolites and pathways, and effects of pathway removal in KFP and rKFP, and some detailed results on the effects of missing data and selecting measuring times. (PDF) [file pcbi.1003958.s001.pdf]

# Estimating Relative Changes of Metabolic Fluxes using Kinetic Flux Profiling

## Text S1

Lei Huang, Dongsung Kim, Xiaojing Liu, Christopher R. Myers and Jason W. Locasale

## Contents

**S0 Supplementary Figures** **2**

**S1 KFP Solution for a Metabolic Pathway** **4**

**S2 Applying (r)KFP to Metabolic Cycles** **8**

**S3 Missing Data and Reduced Models** **11**
S3.1 Metabolite Removal: An Analytical Approach . . . 11
S3.1.1 KFP . . . 11
S3.1.2 rKFP . . . 13
S3.2 Pathway Removal . . . 15
S3.2.1 KFP . . . 15
S3.2.2 rKFP . . . 18
S3.3 How Lumping Isomers Affects KFP . . . 20
S3.4 Plots of costs . . . 21
S3.5 Dynamics of serial and parallel bipartition . . . 22
S3.6 Summary Table . . . 22

**S4 Selecting Measuring Times** **24**
S4.1 Integrating Sensitivity Curves . . . 25
S4.2 Nonconvexity of Minimizing $\sigma_J$ . . . 26
S4.3 The measuring times for $A_1$ . . . 26
S4.4 Calculating $t \approx 0.92\, t_c$ . . . 27
S4.5 Deriving $t = (A_1 + A_2)/J$ for $A_2$ . . . 27
S4.6 Close-to-optimality of $(A_1 + A_2)/J$ for $A_2$ . . . 28
S4.7 Dependence of Estimation Precision on Data Size and Time Selection . . . 28

# S0 Supplementary Figures

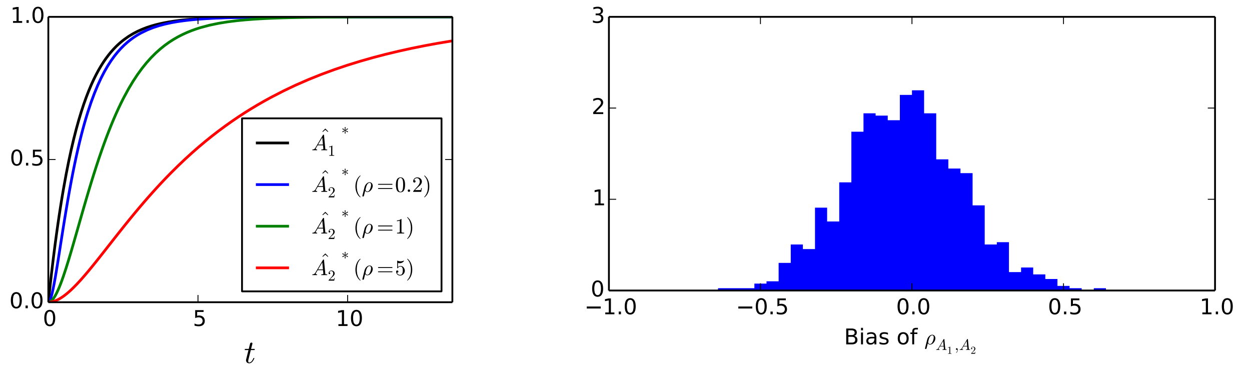


(a) (b)

Figure S1: **Estimating the pool size ratios.** (a) An illustration of how pool size ratio can be inferred from relative quantitation data: after normalization, the more the second metabolite lags behind the first one, the larger its pool size is relative to the first one. (b) A histogram of the bias of $\rho_{A_1,A_2}$, estimated from only relative quantitation of $A_1$ and $A_2$. Parameter values used for generating the simulated data: $A_1 = J = 1$ and $A_2 = 2$. Considering the separation of concentration scale between $A_1$ and $A_2$ can easily be huge in reality (see the last paragraph in the section on selecting measuring times), being able to narrow down the pool size ratio to a 100% interval as the figure shows should be considered an achievement.

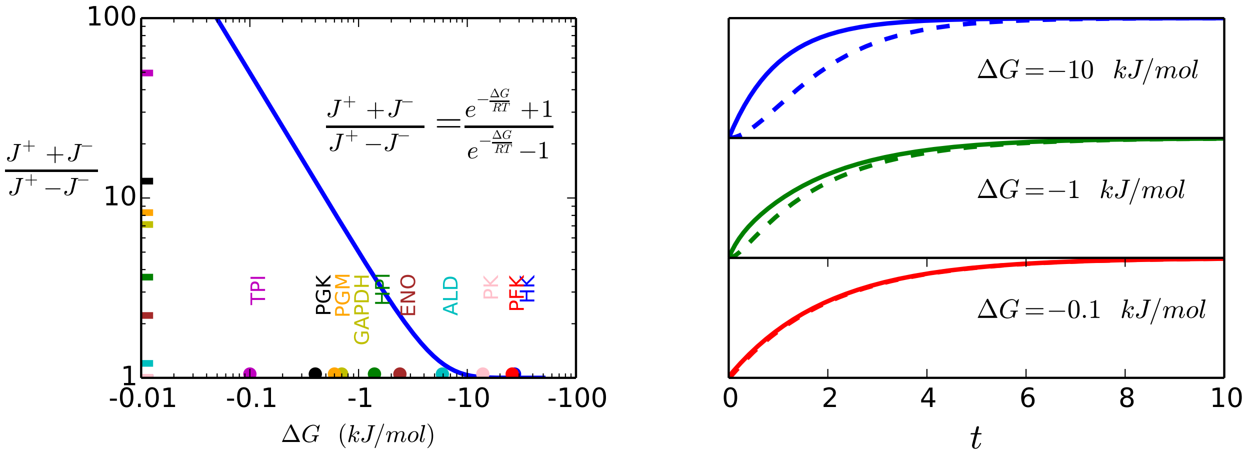


(a) (b)

Figure S2: **The effect of reaction reversibility on KFP.** (a) The dependence of $(J^+ + J^-)/(J^+ - J^-)$ on $\Delta G$. Marked in the plot are the $\Delta G$'s (semicircles) and $(J^+ + J^-)/(J^+ - J^-)$ (bars) of the reactions in glycolysis ($\Delta G$'s from [1]; abbreviations of the reactions: HK: hexokinase; HPI: hexose-phosphate isomerase; PFK: phosphofructokinase; ALD: aldolase; TPI: triose-phosphate isomerase; GAPDH: glyceraldehyde 3-phosphate dehydrogenase; PGK: phosphoglycerate kinase; PGM: phosphoglycerate mutase; ENO: enolase; PK: pyruvate kinase. (b) Convergence of substrate and product pools into one. The solid line represents the $^{13}$C-labeled substrate and the dashed line the $^{13}$C-labeled product.

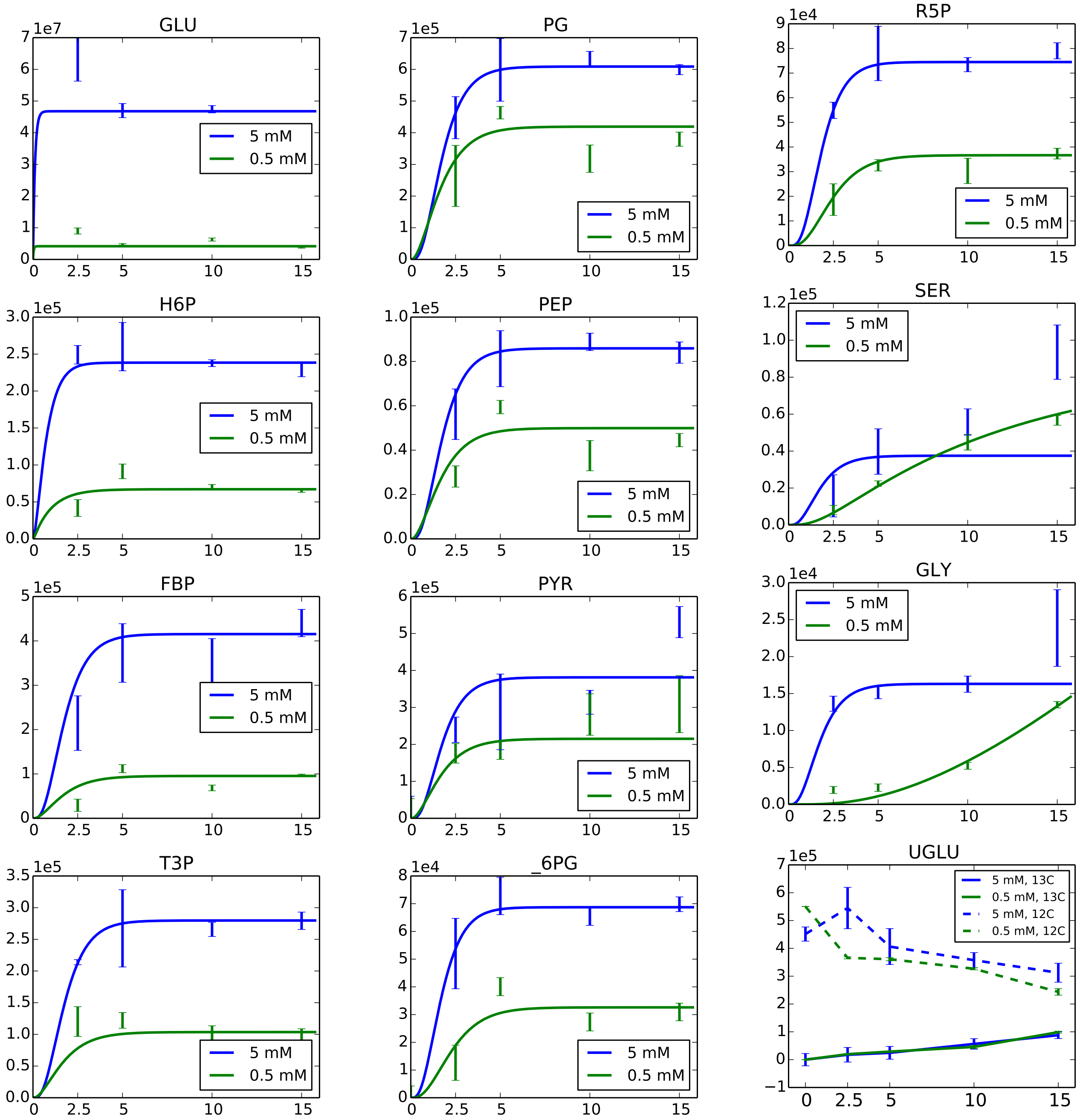


Figure S3: **Analysis of experimental data.** Fitting of experimental data. UGLU is included here to suggest a small flux in glycogen synthesis, which can be seen by the labeled-UGLU slowly approaching the pool size in both conditions.

# S1 KFP Solution for a Metabolic Pathway

In this section, some of the mathematical structure of $^{13}$C-label infiltrating dynamics along a linear metabolic network, or a metabolic pathway, is presented.

Like the toy system in Introduction, the pathway of $n$ metabolites (Figure S4a) is switched from a $^{12}$C environment to a $^{13}$C one at $t = 0$, and the $^{13}$C label will gradually infiltrate the pools of all metabolites along the pathway.

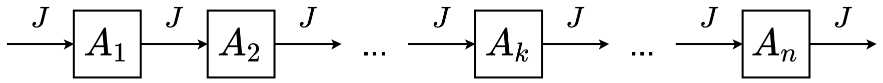


(a)

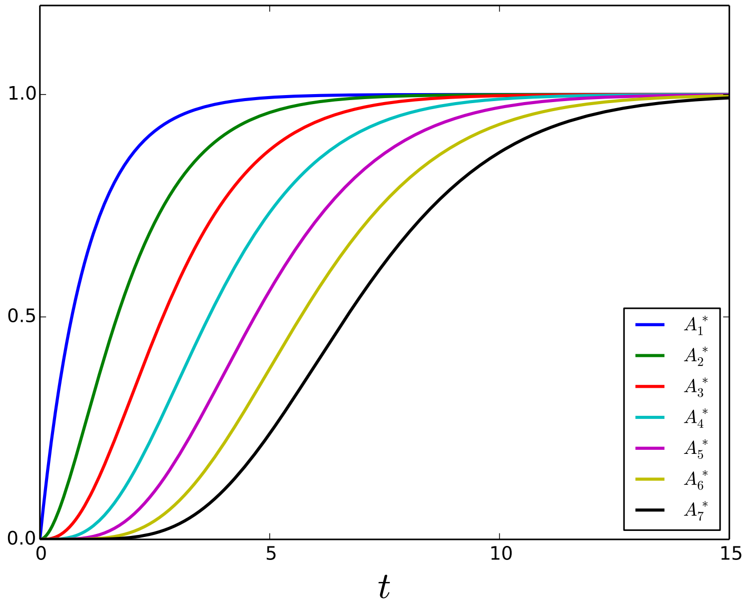


(b)

Figure S4: (a) Diagram of a metabolic pathway of $n$ metabolites. One-dimensional metabolic network. (b) Trajectories of the first seven metabolites. Parameter values for simulating the trajectories: $J = A_k = 1$ $(k = 1, ..., 7)$.

First, one can write down the system of ODEs ($\mu_k \equiv \dfrac{J}{A_k}$):

$$\begin{cases} \dfrac{dA_1^*}{dt} &= J - J\dfrac{A_1^*}{A^1} = J - \mu_1 A_1^*, \\ \dfrac{dA_k^*}{dt} &= J\dfrac{A_{k-1}^*}{A_{k-1}} - J\dfrac{A_k^*}{A_k} = \mu_{k-1}A_{k-1}^* - \mu_k A_k^*, \quad k = 2, ..., n, \end{cases} \tag{S1}$$

which can be written in the matrix form:

$$\frac{d}{dt}\begin{pmatrix} A_1^* \\ A_2^* \\ A_3^* \\ \vdots \\ A_n^* \end{pmatrix} = \begin{pmatrix} -\mu_1 & 0 & 0 & \cdots & 0 & 0 \\ \mu_1 & -\mu_2 & 0 & \cdots & 0 & 0 \\ 0 & \mu_2 & -\mu_3 & \cdots & 0 & 0 \\ \vdots & \vdots & \vdots & \ddots & \vdots & \vdots \\ 0 & 0 & 0 & \cdots & \mu_{n-1} & -\mu_n \end{pmatrix}\begin{pmatrix} A_1^* \\ A_2^* \\ A_3^* \\ \vdots \\ A_n^* \end{pmatrix} + \begin{pmatrix} J \\ 0 \\ 0 \\ \vdots \\ 0 \end{pmatrix}, \text{ or}$$

$$\frac{d\mathbf{A}^*}{dt} = \mathbf{M}\mathbf{A}^* + \mathbf{J} = f(\mathbf{A}^*, \boldsymbol{\theta}), \tag{S2}$$

where $\boldsymbol{\theta} = (\mu_1, \ldots, \mu_n, J)$.

It is a linear system of ODEs with a constant nonhomogeneous term, which can be turned into a linear homogeneous system of ODEs through a translation of $\mathbf{A}^*$. Assuming matrix $\mathbf{M}$ has $n$ distinct

eigenvalues (more below), and using the relevant theory of linear homogeneous systems of ODEs [3], the general solution to Eq. S2 can be written as:

$$A_k^*(t) = \sum_{i=1}^{n} a_i^k e^{\lambda_i t} + c_k, \tag{S3}$$

which says $A_k^*(t)$ is a linear combination of $e^{\lambda_i t}$ plus a translation term, where $\lambda_i$'s are the eigenvalues of matrix $\mathbf{M}$ and $a_i^k$ are arbitrary constants.

Having pinned down the form of the general solution, we can further simplify it using a few considerations:

- Matrix $\mathbf{M}$ is *bidiagonal*, implying that its eigenvalues are its diagonal elements: $\lambda_i = -\mu_i$; then the assumption of distinct eigenvalues translates to distinct $\mu$'s, or distinct $A_i$'s (distinct pool sizes for all metabolites), a generic case in reality.
- By physical intuition, the dynamics of $A_k^*$ should depend only on $J$ and the pool sizes of the metabolites upstream of it, but not downstream, implying $a_i^k = 0$ $(i > k)$.
- $A_k^*(\infty) = A_k$, implying $c_k = A_k$.
- $A_k^*(0) = 0$, implying $\sum_{i=1}^{k} a_i^k = -A_k$.

Therefore, Eq. S3 can be written as:

$$A_k^*(t) = A_k \left(1 - \sum_{i=1}^{k} b_i^k e^{-\mu_i t}\right), \quad \text{where} \quad b_i^k = -a_i^k / A_k \quad \text{and} \quad \sum_{i=1}^{k} b_i^k = 1. \tag{S4}$$

Finally, we use the method of integrating factor to solve for the constant coefficients $b_i^k$'s. Recall that for a first-order linear ODE $y' + f(t)y = g(t)$, an integrating factor is a function $h(t)$ such that, when multiplied to the equation, the left-hand side can be written as $(h(t)y)'$. The resulting ODE of $h(t)$, $h' = f(t)h$, has the solution $h(t) = e^{\int f(t)dt}$, and hence the ODE of $y$ has the solution $y(t) = \frac{1}{h(t)} \int h(t)g(t)dt$ [2].

We rewrite Eq. S1 in the form in the preceding paragraph:

$$\frac{dA_k^*}{dt} + \mu_k A_k^* = \mu_{k-1} A_{k-1}^*, \ (k = 2, ..., n) \tag{S5}$$

and the integrating factor is $h(t) = e^{\mu_k t}$, which gives the following expression of $A_k^*(t)$:

$$A_k^*(t) = \frac{1}{h(t)} \int h(t) \mu_{k-1} A_{k-1}^* dt. \tag{S6}$$

Plugging in the general form of $A_{k-1}^*(t)$ in Eq. S4, we have:

$$\begin{aligned}
A_k^*(t) &= e^{-\mu_k t} \int e^{\mu_k t} \mu_{k-1} A_{k-1} (1 - \sum_{i=1}^{k-1} b_i^{k-1} e^{-\mu_i t}) dt \\
&= J e^{-\mu_k t} \left( \int e^{\mu_k t} dt - \int \sum_{i=1}^{k-1} b_i^{k-1} e^{(\mu_k - \mu_i)t} dt \right) \\
&= J e^{-\mu_k t} \left( \frac{1}{\mu_k} e^{\mu_k t} - \sum_{i=1}^{k-1} b_i^{k-1} \int e^{(\mu_k - \mu_i)t} dt \right) \\
&= J \left( \frac{1}{\mu_k} - e^{-\mu_k t} \sum_{i=1}^{k-1} b_i^{k-1} \frac{1}{\mu_k - \mu_i} \left( e^{(\mu_k - \mu_i)t} + c_i \right) \right).
\end{aligned}$$

Using the initial condition $A_k^*(0) = 0$ allows us to find out the values of $c_i$'s:

$$c_i = -1 + \frac{\mu_k - \mu_i}{b_i^{k-1}\mu_k(k-1)}$$

Plugging the values of $c_i$'s back to the equation:

$$\begin{aligned} A_k^*(t) &= J\left(\frac{1}{\mu_k} - \left(\sum_{i=1}^{k-1} \frac{b_i^{k-1}}{\mu_k - \mu_i}\right) e^{-\mu_i t} - \left(\frac{1}{\mu_k} - \sum_{i=1}^{k-1} \frac{b_i^{k-1}}{\mu_k - \mu_i}\right) e^{-\mu_k t}\right) \\ &= \frac{J}{\mu_k}\left(1 - \left(\left(\sum_{i=1}^{k-1} \frac{\mu_k}{\mu_k - \mu_i} b_i^{k-1}\right) e^{-\mu_i t} + \left(1 - \sum_{i=1}^{k-1} \frac{\mu_k}{\mu_k - \mu_i} b_i^{k-1}\right) e^{-\mu_k t}\right)\right), \end{aligned}$$

and comparing it to the general form of $A_k^*(t) = A_k(1 - \sum_{i=1}^{k} b_i^k e^{-\mu_i t})$, we can conclude:

$$\boxed{\begin{aligned} b_i^k &= \frac{\mu_k}{\mu_k - \mu_i} b_i^{k-1}, \quad i = 1, ..., k-1; \\ b_k^k &= 1 - \sum_{i=1}^{k-1} b_i^k \end{aligned}}$$

Since we know $A_1^*(t) = A_1(1 - e^{-\mu_1 t})$ and hence $b_1^1 = 1$, the boxed equations constitute a recursion rule from which all $b_i^k$'s can be solved.

Example of $A_2^*(t)$:

$$\begin{aligned} b_1^2 &= \frac{\mu_2}{\mu_2 - \mu_1} b_1^1 = \frac{\mu_2}{\mu_2 - \mu_1}, \quad b_2^2 = 1 - b_1^2 = \frac{-\mu_1}{\mu_2 - \mu_1}; \\ A_2^*(t) &= A_2\left(1 - \left(b_1^2 e^{-\mu_1 t} + b_2^2 e^{-\mu_2 t}\right)\right) \\ &= A_2\left(1 - \left(\frac{\mu_2}{\mu_2 - \mu_1} e^{-\mu_1 t} + \frac{-\mu_1}{\mu_2 - \mu_1} e^{-\mu_2 t}\right)\right) \\ &= A_2\left(1 - \left(\frac{A_1}{A_1 - A_2} e^{-\frac{Jt}{A_1}} + \frac{-A_2}{A_1 - A_2} e^{-\frac{Jt}{A_2}}\right)\right). \end{aligned} \tag{S7}$$

Remarks:

1. Eq. S4 says that $A_k^*(t)$ is an exponential approach consisting of an *affine combination* of $k$ exponentials, corresponding to the actions of $k$ metabolite pools from $A_1$ to $A_k$.

2. We use $A_2^*(t)$ to illustrate two detailed features as it is the simplest example of a mixture of exponentials. First, different exponentials in the mixture correspond to the dynamics of different characteristic time-scales, and at the largest characteristic time-scale, the largest contribution to the mixture comes from the slowest exponential; in the case of $A_2^*(t)$, it means at late times, the trajectory is well approximated by the slower exponential corresponding to the action of the larger pool (Figure S5). Second, by Taylor expanding $A_2^*(t)$ at $t = 0$ we find that both the constant and the first-order terms are zero, and the second-order term is $\frac{Jt}{A_1} Jt$, which has the following interpretation: when $t$ is small, the $A_1$ pool mostly takes in $^{13}$C-label without much depleting it, and the proportion of $^{13}$C-label in $A_1$ pool increases approximately linearly in time: $\hat{A_1}^*(t) = \frac{Jt}{A_1}$; the same can be said for the little amount of $^{13}$C in $A_1$ that does move to $A_2$, hence the second-order approximation (Figure S5).

3. The same reasoning in Remark 2 can be applied to other metabolites: $A_k^*(t)$ is approximated by its slowest exponential at late times, and by the polynomial $\prod_{i=1}^{k-1} \frac{Jt}{A_i} Jt$ at early times.

4. It can be shown that $A_k^*(t)$ is invariant with respect to a permutation of the metabolites $A_1$ to $A_{k-1}$: the dynamics of $A_k^*(t)$ depends only on the concentrations of $A_1$ to $A_{k-1}$, but not their ordering (Figure S5). This has an important implication that for a bipartition $q$ of a metabolite pool $A_i$, the dynamics of all $A_j^*(t)$ $(j > i)$ are symmetric in $q$ with respect to $q = 0.5$ (hence the symmetry in Figure S17a).

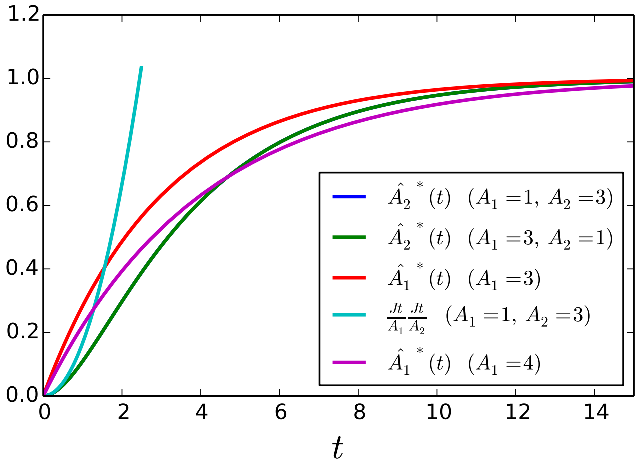


Figure S5: Several normalized trajectories illustrating the dynamics of $A_2^*(t)$. First, both the blue and green curves represent $A_2^*(t)$ and are different in that the pool sizes of $A_1$ and $A_2$ are exchanged; that the two curves overlap suggests the permutation invariance discussed in Remark 4. Second, the red curve represents the dynamics of a single metabolite of the pool size the larger one of $A_1$ and $A_2$, and that it approaches the green curve quickly at late times suggests the domination of the slower exponential in a mixture at late times. Third, the cyan curve represents the second-order approximation to $A_2^*(t)$ at $t = 0$. Finally, the magenta curve represents the dynamics of a single metabolite of pool size the sum of $A_1$ and $A_2$, and in the section on measuring times it suggests that it to a good approximation shares with the green curve when $\partial A^*(t)/\partial J$ is the greatest.

## S2 Applying (r)KFP to Metabolic Cycles

Inspired by the popular concept of "network motifs" from studies of transcriptional networks [5], we try to formulate some basic "architectural motifs" of metabolic networks, and identify three broad classes: linear pathways, branch points, and cycles. Loosely speaking, they carry out biological functions in different ways and have different biological significance: linear pathways are a series of chemical reactions that directly convert raw materials into desired products, while cycles serve as "conveyor belts" that perform the conversion between materials that themselves are not directly enzymatically linked; branch points serve as decision points for channeling the materials into different fates and uses [6].

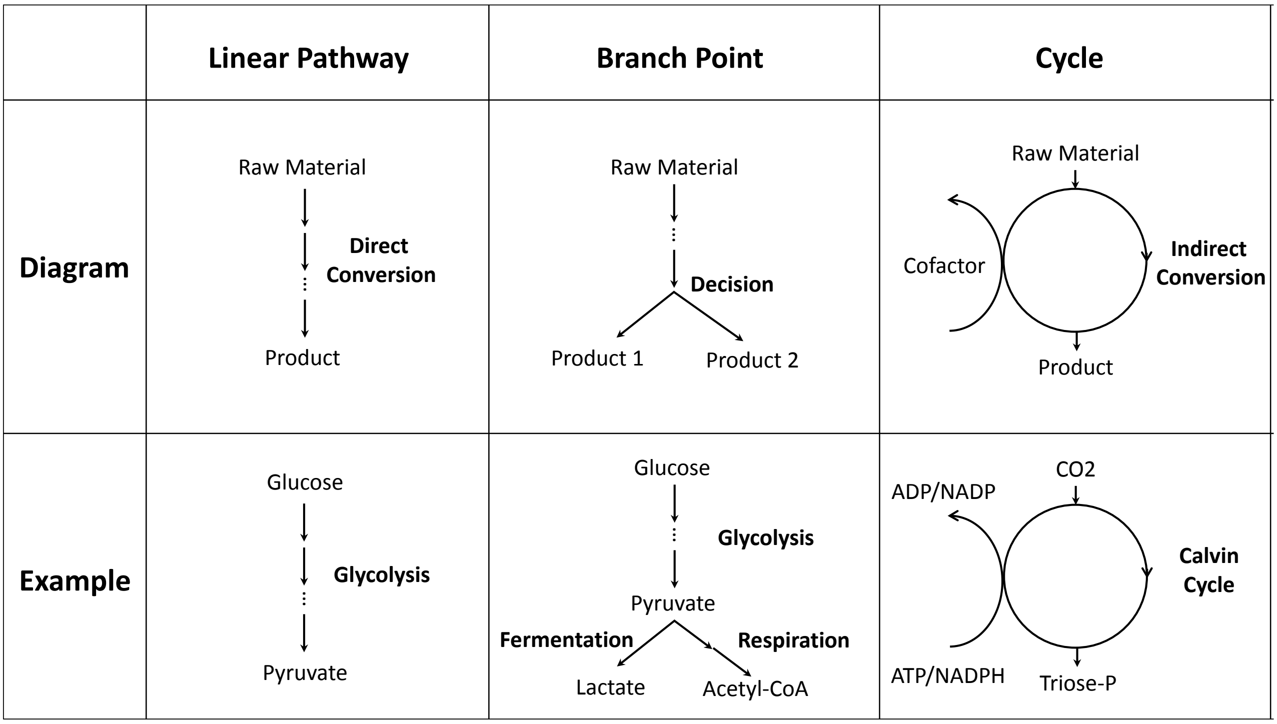


Figure S6: "Architectural motifs" of metabolic networks.

The main text has described and demonstrated the capacity of KFP and rKFP for estimating the fluxes in linear pathways and branch points. It behooves us to discuss the remaining case of cycles, the subject of this section.

We first observe that, as a result of the fundamental "conveyor belt" design of cycles, they always involve reactions that have more than one substrate, and these reactions are of two kinds: those involving cofactors and those around the entry point of the cycles (such as the reactions catalyzed by citrate synthase in TCA cycle and RuBisCO in Calvin Cycle). For multi-substrate reactions involving the common cofactors such as ATP/ADP and $NADH/NAD^+$, the reaction equations are of the forms $A+ATP \longleftrightarrow AP + ADP$ or $A+NADH+H^+ \longleftrightarrow B + NAD^+$, where cofactors do not participate in the shuffling of the carbon backbones of the substrates. This is in contrast to multi-substrate reactions at the entry point of the cycles, which shuffle the carbon backbones of the substrates and pose challenges to KFP and rKFP modeling, as we explain below.

Take citrate synthase as an example. The reaction equation is acetyl-CoA + oxaloacetate $\longleftrightarrow$ citrate + CoA, where the two-carbon acetyl group of acetyl-CoA is attached to the four-carbon oxaloacetate to form the six-carbon citrate. We claim that (r)KFP as it currently stands cannot handle reactions where the carbons of a product have more than one source, such as citrate in this case. First, when there are two sources of carbons for a product, it can have multiple labeling states: for example, fully labeled acetyl-CoA reacts with unlabeled oxaloacetate to form citrate labeled in two carbons, while unlabeled acetyl-CoA reacts with fully labeled oxaloacetate to form citrate labeled in four carbons; this deviates the assumption in (r)KFP that a metabolite is either labeled or unlabeled (hence our notation of $A^*$ for the labeled state). Second, a full description of all the labeling states of a metabolite and their dynamics requires the knowledge of carbon transition

between reactions: for example, the carbon transition map of citrate synthase can be represented as ①②-CoA + ③④⑤⑥ $\longleftrightarrow$ ②①④⑤⑥③ + CoA, where the circles represent carbon atoms and the numbering keeps track of their transition in a reaction; in comparison the transitions of labeled to labeled and unlabeled to unlabeled as considered by (r)KFP are much simpler. It is worth noting that most biochemical reactions involve more than one substrate [7]; moreover, if a reaction involving one substrate but multiple products (a cleavage reaction) is reversible, which is technically always true and practically usually so, then the reverse reaction would be of a multi-substrate kind. Therefore, this nuisance case in the eyes of (r)KFP is actually a general one.

To model the carbon transition of these multi-substrate reactions, a natural approach is to expand the state variables to incorporate all possible labeling states and model their dynamics by using the knowledge of carbon transitions of the relevant reactions [8]; this approach is equivalent to a kinetic version of metabolic flux analysis (kMFA) [9], as MFA traditionally considers only steady-state [10]. In short, kMFA reduces to KFP when there are only two labeling states, and reduces to MFA when restricted to steady-state. Note that when more than two labeling states have to be considered, generally the state space and model complexity are huge, and efforts of simplifying them are involved [12].

Fortunately, Szecowka *et al.* proposed a clever variant of KFP [11] that is able to handle multi-substrate reactions, which they term extended KFP, or eKFP. The method allows them to estimate the fluxes of Calvin Cycle in *Arabidopsis* without having to deal with the complexity of (k)MFA. Below we briefly describe their method, and apply the rKFP version of it (which we term reKFP) to estimate relative flux changes of a simplified TCA cycle using simulated data, demonstrating the capacity of (r)KFP for metabolic cycles.

It is mentioned above that when there are multiple carbon sources for a product in a reaction, it has multiple labeled states and keeping track of their dynamics requires the knowledge of carbon transitions of the reaction. The central idea of eKFP is that in contrast there is always only one unlabeled state and its dynamics is always simple and requires no knowledge of atom transition. Consider again the example of citrate synthase: there is only one unlabeled state of citrate (unlabeled in all six carbons) and the only way to generate this state in the reaction is unlabeled acetyl-CoA reacting with unlabeled oxaloacetate. Therefore, instead of modeling the labeled states as in (r)KFP, eKFP models only the unlabeled states, and bypasses all the complexity entailed by the carbon scrambling in a multi-substrate reaction. Clever as it is, the approach has two drawbacks: conceptually, much information is lost by discarding all data of the labeled metabolites; practically, measurements of unlabeled metabolites are often inflated by an unknown amount due to the contamination of media in the sample. Using simulated data below that suffer no media contamination, we adopt the idea of eKFP and combine it with rKFP; the resulting reKFP is shown to be applicable to cycles.

Figures S7a and S7b show the diagrams of the metabolic networks used in our simulation: they are adapted from a pioneering and systematic study of fluxomic changes before and after viral infection [13], simplified to some extent but still maintaining the crucial features including the TCA cycle; the numbers of pool sizes and fluxes are adapted from the study as well, from which simulated data are generated. Applying reKFP to the simulated data reliably uncovers the relative flux changes used in the simulation (Figure S7c).

To summarize, (r)KFP can be applied to metabolic cycles by using the idea of modeling only the unlabeled state from eKFP. The resulting method is less powerful than a full-blown kMFA since it uses only part of the data, but enjoys a tremendous reduction of model complexity. In modeling the data of unlabeled metabolites, one has to be careful about the bias introduced by the media to the sample.

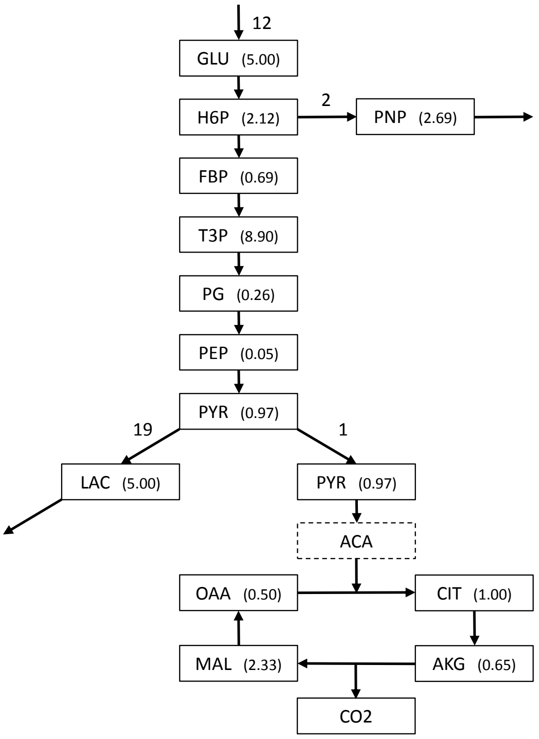


(a) Control.

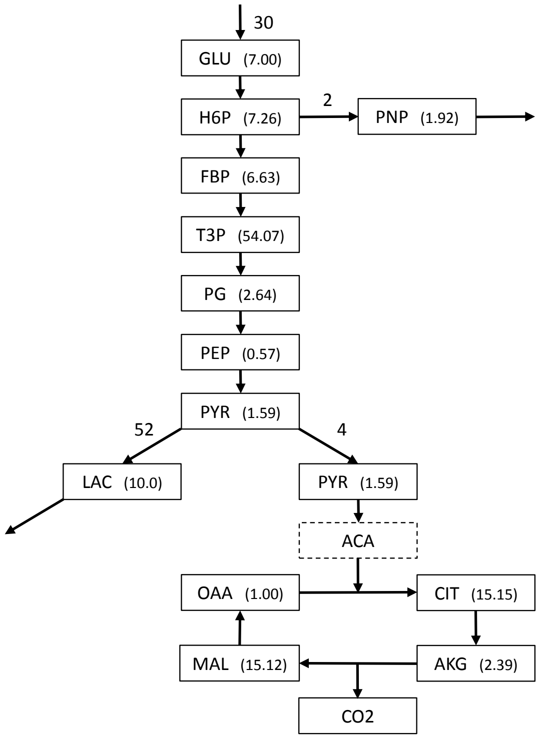


(b) Virus-infected.

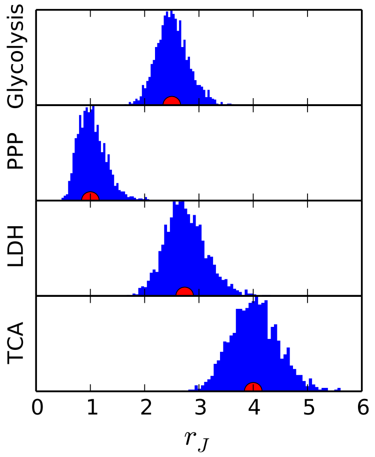


(c) Estimation results.

Figure S7: The two metabolic networks are adapted from [13], corresponding to before and after viral infection studied in the paper respectively. The numbers in parentheses near metabolites denote pool sizes, and numbers near reaction arrows denote fluxes, also adapted from [13]. The dashed box around ACA denotes missing data and PYR in cytosol and mitochondria share one single pool, both in accordance with the study. The red dots in Panel (c) denote the values of $r_J$ used in the simulation. Measuring times for generating simulated data are set to be 1, 2, 3, 5, 10, 15 and 20 minutes. Abbreviation schemes: H6P: hexose-6-phosphate (G6P+F6P); T3P: triose-3-phosphate (DHAP+GAP); PG: phosphoglycerate (3PG+2PG); PNP: pentose-phosphate; ACA: acetyl-CoA; CIT: citrate; AKG: alpha-ketoglutarate; MAL: malate; OAA: oxaloacetate.

# S3 Missing Data and Reduced Models

## S3.1 Metabolite Removal: An Analytical Approach

In the main text, we describe how metabolite removal affects both KFP and rKFP using a computational approach: mimicking the practical situation, a few time points along the trajectories are selected to constitute data used for estimating parameters. In this section, we describe a complementary approach that allows for analytical insight. We first describe in details how it is applied to metabolite removal in KFP, and sketch the results for metabolite removal in rKFP.

### S3.1.1 KFP

Figure S8a sketches a cartoon of metabolite removal in KFP; it is made even simpler than the one in the main text to be analytically tractable: for a linear pathway of two metabolites, the first one is hard to measure and therefore removed in the reduced model. The metabolite that provides data and is used for fitting is hence just the second metabolite $A_2$, and Figure S8b plots its trajectories in both the full and reduced models: in the full model, due to the slowing-down of the spreading of $^{13}$C label by the $A_1$ pool, $A_2^*$ has an initial lag (Figure S5), whereas in the reduced model since $A_1$ is absent, $A_2^*$ (or $\tilde{A}_2^*$ in this case; $\tilde{}$ denotes quantities in the reduced model) is simply the exponential approaching function. Paramterized by the fitting parameter $\tilde{J}$, $\tilde{A}_2^*$ tries to fit $A_2^*$ as well as it can; in the computational approach, the goodness of fit is measured by its closeness to $A_2^*$ at a number of discrete time points, whereas an alternative measure for the goodness-of-fit is the area between the two curves (the shaded region in light red): the greater the area of the region, the poorer the fit. To further extend the analytical tractability, instead of the area which would be $\int_0^\infty |\tilde{A}_2^* - A_2^*| \mathrm{d}t$ (technically the distance between $\tilde{A}_2^*$ and $A_2^*$ using $L^1$ norm), we use the square of the difference: $\int_0^\infty (\tilde{A}_2^* - A_2^*)^2 \mathrm{d}t$ (technically the distance between $\tilde{A}_2^*$ and $A_2^*$ using $L^2$ norm), called the cost of the fitting which is a function of $\tilde{J}$, $C(\tilde{J})$, and the goal is to find $\tilde{J}$ that minimizes $C(\tilde{J})$.

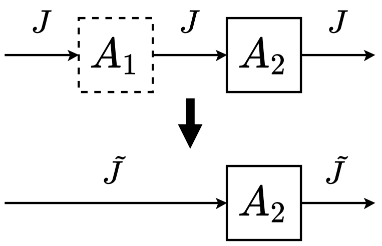


(a) Diagram of the models used in the analysis.

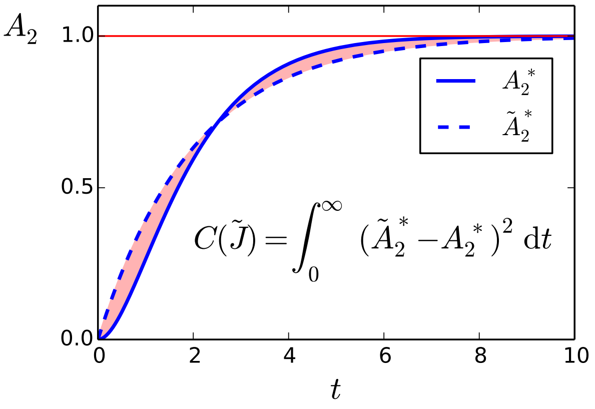


(b) Parameter values used for the simulation: $A_1 = A_2 = J = 1$.

Figure S8: Metabolite removal in KFP.

Before we proceed to describe the method, we note that using as the definition of $C(\tilde{J})$ the integral of squared differences over a continuous region, rather than the sum of squared differences over a discrete set of points, has two immediate advantages. First, it does not require selecting a set of measuring times and makes comparing estimation results using different parameters much easier (one of the uses of the method of selecting measuring times described in the main text is to enable such comparison). Second, since the integration is from the origin to infinity, it requires that both $\tilde{A}_2^*$ and $A_2^*$ have the same saturation level $A_2$ (the red line in Figure S8b) (otherwise $C(\tilde{J})$ would be infinite), and enables us to focus on the comparison of $J$.

To continue investigating how metabolite removal affects KFP using the analytical approach, we first write down the formula for $\tilde{A}_2^*$ and $A_2^*$. Section S1 gives that:

$$\begin{aligned} A_2^*(t) &= A_2\left(1-\left(\frac{A_1}{A_1-A_2}e^{-\frac{Jt}{A_1}}+\frac{-A_2}{A_1-A_2}e^{-\frac{Jt}{A_2}}\right)\right);\\ \tilde{A}_2^*(t) &= A_2\left(1-e^{-\frac{\tilde{J}t}{A_2}}\right). \end{aligned}$$

They give $C(\tilde{J})$:

$$\begin{aligned} C(\tilde{J}) &= \int_0^\infty (\tilde{A}_2^*-A_2^*)^2 \mathrm{d}t\\ &= \int_0^\infty \left(A_2\left(1-e^{-\frac{\tilde{J}t}{A_2}}\right)-A_2\left(1-\left(\frac{A_1}{A_1-A_2}e^{-\frac{Jt}{A_1}}+\frac{-A_2}{A_1-A_2}e^{-\frac{Jt}{A_2}}\right)\right)\right)^2 \mathrm{d}t\\ &= A_2^2\int_0^\infty \left(\left(\frac{A_1}{A_1-A_2}e^{-\frac{Jt}{A_1}}+\frac{-A_2}{A_1-A_2}e^{-\frac{Jt}{A_2}}\right)-e^{-\frac{\tilde{J}t}{A_2}}\right)^2 \mathrm{d}t \end{aligned}$$

$$\begin{aligned} = A_2^2\Bigg(&\left(\frac{A_1}{A_1-A_2}\right)^2\frac{1}{\frac{2J}{A_1}}+2\frac{A_1}{A_1-A_2}\frac{-A_2}{A_1-A_2}\frac{1}{\frac{J}{A_1}+\frac{J}{A_2}}+\left(\frac{-A_2}{A_1-A_2}\right)^2\frac{1}{\frac{2J}{A_2}}-\\ &2\left(\frac{A_1}{A_1-A_2}\frac{1}{\frac{J}{A_1}+\frac{\tilde{J}}{A_2}}+\frac{-A_2}{A_1-A_2}\frac{1}{\frac{J}{A_2}+\frac{\tilde{J}}{A_2}}\right)+\frac{1}{\frac{2\tilde{J}}{A_2}}\Bigg) \end{aligned}$$

To find $\tilde{J}$ at which $C(\tilde{J})$ is minimum, we differentiate $C(\tilde{J})$ with respect to $\tilde{J}$ and set it to zero:

$$\frac{\mathrm{d}C(\tilde{J})}{\mathrm{d}\tilde{J}} = 0. \tag{S8}$$

We define two new dimensionless variables:

$$\begin{aligned} \rho &\equiv \frac{A_1}{A_2}, &&\text{pool size ratio (note that } \rho \equiv \frac{A_2}{A_1} \text{ in the main text);}\\ \beta &\equiv \frac{\tilde{J}-J}{J}, &&\text{bias (defined in the main text).} \end{aligned}$$

Substituting them into Eq. S8 and simplifying the equation we get:

$$3\rho^2\beta^4+6\rho(1+3\rho)\beta^3+3(1+6\rho+13\rho^2)\beta^2+4(1+4\rho+9\rho^2)\beta+4\rho(1+3\rho)=0 \tag{S9}$$

We pause here and highlight an important observation: after simplification no variables with units such as $J$ or $A_2$ remain in Eq. S9, which means that the functional relationship as defined by Eq. S9 between the two dimensionless variables, $\rho$ and $\beta$, is an intrinsic one and does not depend on the scale: the *relative* bias in the estimated $J$ depends *only* on the *relative* pool size difference between the two metabolites. This also justifies one aspect of the computational approach used in the main text: there some values of $J$ and $A$'s are used in the simulation and here it says that the results there hold regardless what values are chosen for the parameters as long as their relative ratios are properly controlled which is the case.

Figure S9 plots the functional relationship between $\beta$ and $\rho$ and summarizes the result: $\beta$ decreases from 0 as $\rho$ increases from 0; in other words, there is little bias when the $A_1$ pool size is negligibly small *compared to* $A_2$, and $J$ is increasingly underestimated when the $A_1$ pool size relative to $A_2$ increases, all making sense given the intuition described in the main text.

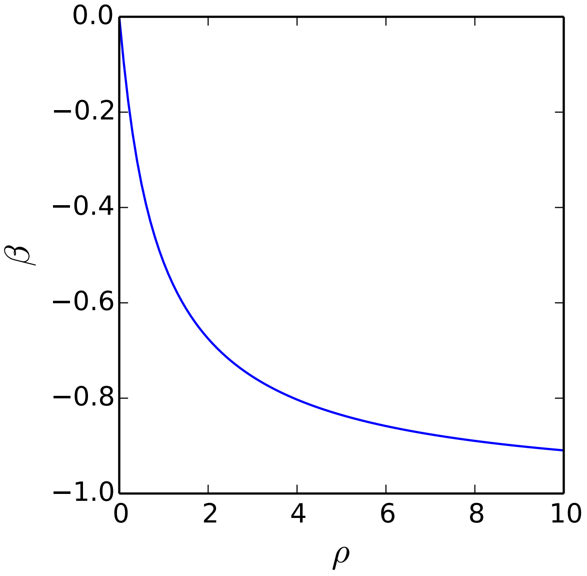


Figure S9: Dependence of bias $\beta$ on pool size ratio $\rho$.

### S3.1.2 rKFP

To apply the same analytical approach to metabolite removal in rKFP (a diagram of the reduction is provided in Figure S10a), we first write down the equations for $a_2^*$ in both conditions in the full model:

$$a_{2x}^*(t) = a_2\left(1 - \left(\frac{A_1}{A_1 - A_2}e^{-\frac{Jt}{A_1}} + \frac{-A_2}{A_1 - A_2}e^{-\frac{Jt}{A_2}}\right)\right) = a_2\left(1 - \left(\frac{A_1}{A_1 - A_2}e^{-\mu_1 t} + \frac{-A_2}{A_1 - A_2}e^{-\mu_2 t}\right)\right);$$

$$\begin{aligned} a_{2y}^*(t) &= a_2\left(1 - \left(\frac{r_1 A_1}{r_1 A_1 - r_2 A_2}e^{-\frac{RJt}{r_1 A_1}} + \frac{-r_2 A_2}{r_1 A_1 - r_2 A_2}e^{-\frac{RJt}{r_2 A_2}}\right)\right) \\ &= a_2\left(1 - \left(\frac{r_1 A_1}{r_1 A_1 - r_2 A_2}e^{-\frac{R\mu_1 t}{r_1}} + \frac{-r_2 A_2}{r_1 A_1 - r_2 A_2}e^{-\frac{R\mu_2 t}{r_2}}\right)\right), \end{aligned}$$

where $r_1$ and $r_2$ and $R$ are the relative changes for the pool sizes $A_1$, $A_2$ and flux $J$ respectively, and $\mu_1 \equiv J/A_1$ and $\mu_2 \equiv J/A_2$ are the rates. The corresponding equations in the reduced model are:

$$\tilde{a}_{2x}^*(t) = a_2\left(1 - e^{-\frac{\tilde{J}t}{\tilde{A}_2}}\right) = a_2\left(1 - e^{-\tilde{\mu}_2 t}\right);$$

$$\tilde{a}_{2y}^*(t) = a_2\left(1 - e^{-\frac{\tilde{R}\tilde{J}t}{r_2\tilde{A}_2}}\right) = a_2\left(1 - e^{-\frac{\tilde{R}\tilde{\mu}_2 t}{r_2}}\right),$$

where quantities $a_2$ and $r_2$ are the same as those in the full model as forced by the cost of integrating from 0 to $\infty$, while quantities $\mu_2$ and $R$ are potentially different from those in the full model (as denoted by $\tilde{}$). The cost is hence:

$$C(\tilde{\mu_2}, \tilde{R}) = \int_0^\infty \left((\tilde{a}_{2x}^* - a_{2x}^*)^2 + (\tilde{a}_{2y}^* - a_{2y}^*)^2\right) \mathrm{d}t,$$

which evaluates to a complicated expression that we neglect here. We proceed in similar steps as in the KFP case: differentiating $C(\tilde{\mu_2}, \tilde{R})$ with respect to $\tilde{\mu_2}$ and $\tilde{R}$, setting them to zero, making substitutions $\rho_A \equiv \dfrac{A_1}{A_2}$, $\rho_r \equiv \dfrac{r_1}{r_2}$, $\beta_\mu \equiv \dfrac{\tilde{\mu_2} - \mu_2}{\mu_2}$ and $\beta_R \equiv \dfrac{\tilde{R} - R}{R}$, and simplifying the equations. Finally we reach two equations that define a function that maps from $(\rho_A, \rho_r)$ to $(\beta_\mu, \beta_R)$:

$$
\begin{aligned}
&(\beta_\mu+2)^2(\rho_A(\beta_\mu+1)+1)^2\big(3\rho_A^2(\beta_\mu+1)^2\rho_r^2(\beta_R+1)^2(\beta_\mu+(\beta_\mu+1)\beta_R+2)^2\\
&\quad+2\rho_A(\beta_\mu+1)\rho_r(\beta_R+1)\big(3\beta_\mu(\beta_\mu+2)+3(\beta_\mu+1)^2\beta_R^2+6(\beta_\mu+1)^2\beta_R+2\big)\\
&\quad+3\beta_\mu^2+4\beta_\mu+3\beta_\mu^2\beta_R^2+6\beta_\mu\beta_R^2+6\beta_\mu^2\beta_R+10\beta_\mu\beta_R+3\beta_R^2+4\beta_R\big)\\
&\quad+(\beta_R+1)(\beta_\mu+(\beta_\mu+1)\beta_R+2)^2\big(3\rho_A^2(\beta_\mu+1)^2(\beta_\mu+2)^2\\
&\quad+2\rho_A(\beta_\mu+1)(3\beta_\mu(\beta_\mu+2)+2)+\beta_\mu(3\beta_\mu+4)\big)(\rho_A(\beta_\mu+1)\rho_r(\beta_R+1)+1)^2=0;
\end{aligned}
$$

$$
\begin{aligned}
&3\rho_A^2(\beta_\mu+1)^2\rho_r^2(\beta_R+1)^2(\beta_\mu+(\beta_\mu+1)\beta_R+2)^2\\
&\quad+2\rho_A(\beta_\mu+1)\rho_r(\beta_R+1)\Big(3\beta_\mu(\beta_\mu+2)+3(\beta_\mu+1)^2\beta_R^2+6(\beta_\mu+1)^2\beta_R+2\Big)\\
&\quad+3\beta_\mu^2+4\beta_\mu+3\beta_\mu^2\beta_R^2+6\beta_\mu\beta_R^2+6\beta_\mu^2\beta_R+10\beta_\mu\beta_R+3\beta_R^2+4\beta_R=0.
\end{aligned}
$$

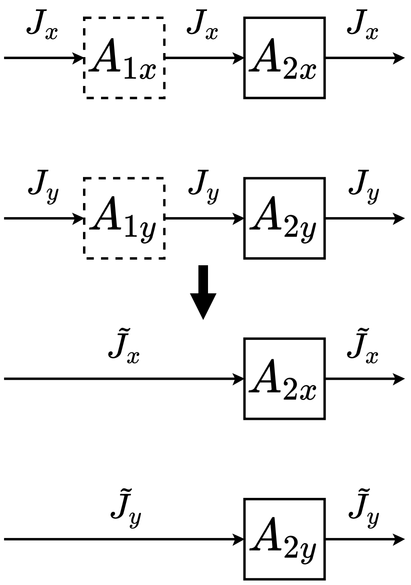


(a) Diagram of the models used in the analysis.

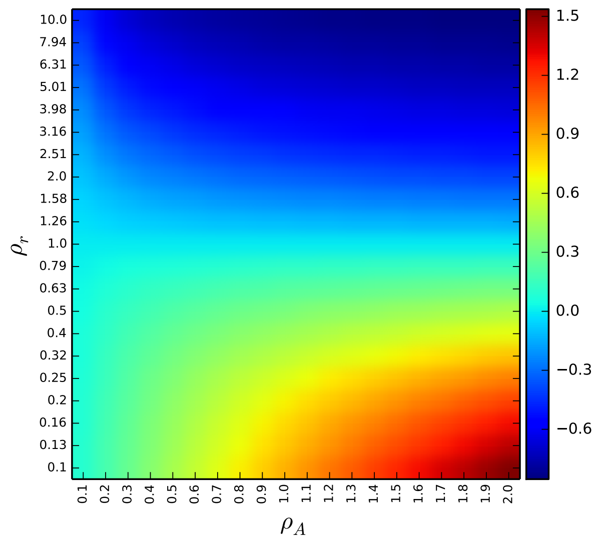


(b) Bias of $R$ ($\beta_R$). Note that $\beta_R = 0$ when $\rho_r = 1$, and its similarity to Figure 3b in the main text.

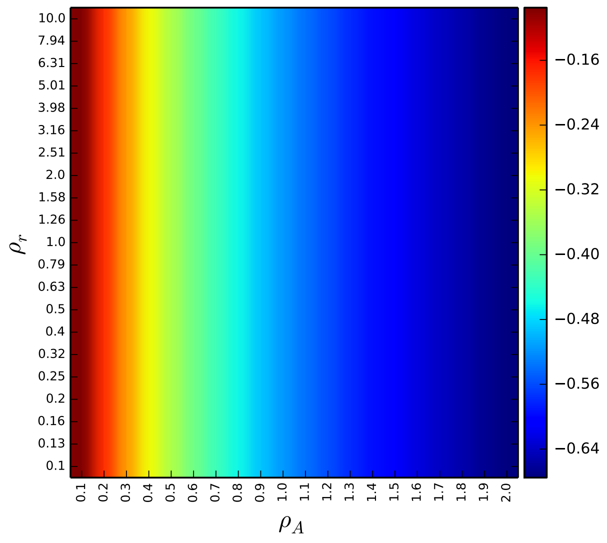


(c) Bias of $\mu_2$ ($\beta_\mu$).

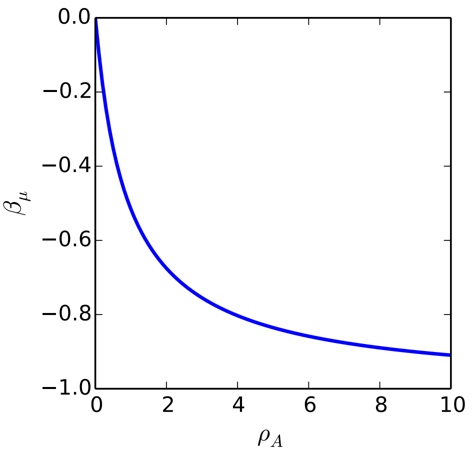


(d) The dependence of $\beta_\mu$ on $\rho_A$ alone. Note that it is the same as Figure S9.

Figure S10: Metabolite removal in rKFP.

Figure S10b plots the functional dependence of $\beta_R$ on $\rho_A$ and $\rho_r$. To interpret it, it is worthwhile examining the definition of $\rho_r$ again: $\rho_r \equiv \frac{r_1}{r_2}$, which is the ratio of relative pool size changes between $A_1$ and $A_2$. A potentially more illuminating interpretation of the quantity can be obtained by rewriting it as $\rho_r \equiv \frac{r_1}{r_2} = \frac{\frac{r_1 A_1}{A_1}}{\frac{r_2 A_2}{A_2}} = \frac{\frac{r_1 A_1}{r_2 A_2}}{\frac{A_1}{A_2}} = \frac{\rho_{Ay}}{\rho_{Ax}}$, where $\rho_{Ax}$ and $\rho_{Ay}$ are the pool size ratios between the two metabolites in the two conditions respectively ($\rho_{Ax}$ is written as $\rho_A$ for short hitherto); in other words, $\rho_r$ is also the ratio in the relative pool size comparison of the two metabolites between the two conditions. Figure S10b shows that $\beta_R = 0$ when $\rho_r = 1$, or, in words, when the two metabolite pools change by the same relative amount between the two conditions, which leads to the same pool size ratios between the two metabolites in the two conditions, there is no bias in the estimated relative flux change. Combined with the result in KFP (*i.e.*, the relative pool size ratio between the two metabolites determines the bias in the estimated flux), that $\beta_R = 0$ when $\rho_r = 1$ can be interpreted as that there is no bias in the relative flux change relative because the biases introduced separately to the estimated fluxes in the two conditions cancel out due to the same relative pool size ratios in the two conditions. Having understood this, the rest of the figure is straightforward to interpret: when $\rho_r > 1$, the relative pool size ratio in the condition ($\rho_{Ay}$) is greater than in the control ($\rho_{Ax}$), causing more downward bias in the condition than in the control, hence causing $R$ to be underestimated, and vice versa when $\rho_r < 1$; the bias in $R$ get more severe as $\rho_A$ increases due to the nonlinear dependence of $\beta_J$ on $\rho_A$ in KFP (Figure S9). Roughly speaking, $\rho_r$ determines the sign of $\beta_R$, and $\rho_A$ determines the magnitude.

Figure S10c plots the dependence of $\beta_\mu$, the bias in the estimated rate, on $\rho_A$ and $\rho_r$, which shows the lack of dependence on $\rho_r$; replotting the dependence on $\rho_A$ only gives Figure S10d, which is of exactly the same shape as Figure S9. Combined with our interpretation of the dependence of $\beta_R$ above, this suggests an important idea on understanding rKFP: when it comes to the bias in rKFP, it might be conceptually *decomposable* into two separate KFPs, examining the bias introduced in each of them, and combining them afterwards. We have verified this idea to be working in the case of metabolite removal, hence significantly simplifying our understanding of the problem. However, numerical experiments show that it is not completely working in the case of pathway removal (agree only roughly); it is an interesting open problem in what cases an rKFP is decomposable to two KFPs in terms of bias.

## S3.2 Pathway Removal

Another common reduction is to remove branching pathways with poor data coverage: if a branching pathway has few metabolites reliably measured, one feels tempted to leave it out. Here we investigate the bias introduced in this way, and the conditions for the bias to be small.

### S3.2.1 KFP

Figure S11a draws a cartoon of the reduction in KFP: two pathways branch out of $A_1$ and one of them has poor data coverage (*e.g.*, $A_2$ is poorly measured), and therefore a reduced model with the pathway removed is used for estimating $J$. Figure S11b illustrates the consequence of using the reduced model: given $J_1 = \tilde{J}$, in the reduced model where there is no flux leaking out of $A_1$ to $A_2$, all flux goes to $A_3$ which makes $\tilde{A}_3^*$ saturate faster than $A_3^*$ in the full model, while $\tilde{A}_1^*$ is the same as $A_1^*$; therefore, in order to fit to the actual slow-saturating $A_3^*$, the reduced model has to lower $\tilde{J}$ to slow down $\tilde{A}_3^*$, causing underestimation of $J$.

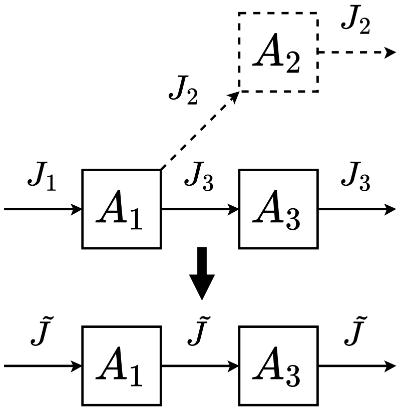


(a) Diagram of the models used in the analysis.

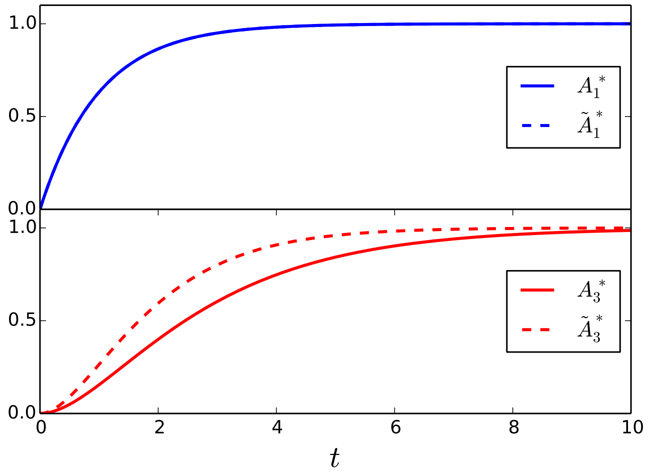


(b) Parameter values used in the simulation: $A_1 = A_2 = A_3 = J_1 = \tilde{J} = 1, J_2 = J_3 = 0.5$.

Figure S11: Pathway removal in KFP.

We now apply the analytical approach to the reduction. First we write down the equations for the variables used as data in both the full and reduced models:

$$
\begin{aligned}
A_1^* &= A_1\left(1 - e^{-\frac{J_1 t}{A_1}}\right),\\
A_3^* &= A_3\left(1 - \left(\frac{\frac{J_3}{A_3}}{\frac{J_3}{A_3} - \frac{J_1}{A_1}} e^{-\frac{J_1 t}{A_1}} + \frac{-\frac{J_1}{A_1}}{\frac{J_3}{A_3} - \frac{J_1}{A_1}} e^{-\frac{J_3 t}{A_3}}\right)\right);\\
\tilde{A}_1^* &= A_1\left(1 - e^{-\frac{\tilde{J} t}{A_1}}\right),\\
\tilde{A}_3^* &= A_3\left(1 - \left(\frac{\frac{\tilde{J}}{A_3}}{\frac{\tilde{J}}{A_3} - \frac{\tilde{J}}{A_1}} e^{-\frac{\tilde{J} t}{A_1}} + \frac{-\frac{\tilde{J}}{A_1}}{\frac{\tilde{J}}{A_3} - \frac{\tilde{J}}{A_1}} e^{-\frac{\tilde{J} t}{A_3}}\right)\right).
\end{aligned}
$$

Next we substitute in the rate variables to simplify the equations: $\mu_1 \equiv \dfrac{J_1}{A_1}$, $\mu_3 \equiv \dfrac{J_3}{A_3}$, $\tilde{\mu}_1 \equiv \dfrac{\tilde{J}}{A_1}$ and $\tilde{\mu}_3 \equiv \dfrac{\tilde{J}}{A_3}$. Then one can write down the cost as a function of $\mu_1, \mu_3, \tilde{\mu}_1, \tilde{\mu}_3, A_1$ and $A_3$:

$$
C(\mu_1, \mu_3, \tilde{\mu}_1, \tilde{\mu}_3, A_1, A_3) = \int_0^\infty \left((\tilde{A}_1^* - A_1^*)^2 + (\tilde{A}_3^* - A_3^*)^2\right) \mathrm{d}t.
$$

Once the cost is evaluated, the expression of which we omit here due to its complexity, one can make two more substitutions to nondimensionalize the variables: $\rho_A \equiv \dfrac{A_3}{A_1}$ and $\rho_J \equiv \dfrac{J_3}{J_1}$, and the resulting expression of the cost becomes a function of $\mu_1, \tilde{\mu}_1, \rho_A$ and $\rho_J$. Setting the derivative of the cost with respect to $\tilde{\mu}_1$ to zero, which is equivalent to setting its derivative with respect to $\tilde{J}$ to zero as $\tilde{J}$ and $\tilde{\mu}_1$ are different by a constant $A_1$, and making one final substitution to introduce the bias variable: $\beta \equiv \dfrac{\tilde{J} - J_1}{J_1}$, we get an equation of $\beta, \rho_A$ and $\rho_J$ (note that like the case in metabolite removal, the variable with dimension, $\mu_1$, drops out after simplification), which defines a function mapping from $(\rho_A, \rho_J)$ to $\beta$:

$$
\begin{aligned}
&\left(\beta^2+3\beta+2\right)^2\rho_A^8\left(3(\beta+1)^2-2(\beta+1)\rho_J-\rho_J^2\right)\\
&+(\beta+1)(\beta+2)^2\rho_A^7\left((6\beta+11)(\beta+1)^3-\left(2\beta^2+11\beta+9\right)\rho_J^2-2\rho_J^3\right)\\
&+(\beta+2)^2\rho_A^6\left(\left(3\beta^2+16\beta+16\right)(\beta+1)^4+\left(3\beta^2-17\right)(\beta+1)^2\rho_J^2\right.\\
&\left.-4\left(\beta^2+4\beta+3\right)\rho_J^3+2(3\beta+7)(\beta+1)^4\rho_J-\rho_J^4\right)\\
&+\beta\left(3\beta^2+7\beta+4\right)\rho_A\rho_J\left(\beta+\rho_J+1\right)^2\left(2(\beta+1)^2+(\beta+3)\rho_J\right)\\
&+\rho_A^5\left(\left(5\beta^4+36\beta^3+98\beta^2+112\beta+44\right)(\beta+1)^4\right.\\
&+4\left(4\beta^4+26\beta^3+61\beta^2+59\beta+19\right)(\beta+1)^3\rho_J\\
&+\left(17\beta^4+90\beta^3+141\beta^2+44\beta-32\right)(\beta+1)^2\rho_J^2\\
&\left.+2\left(3\beta^5+15\beta^4+13\beta^3-37\beta^2-72\beta-34\right)\rho_J^3-(\beta+2)^2(2\beta+5)\rho_J^4\right)\\
&+\rho_A^4\left(\beta+\rho_J+1\right)^2\left(\left(3\beta^4+24\beta^3+56\beta^2+48\beta+12\right)(\beta+1)^2\right.\\
&\left.+\left(3\beta^4+12\beta^3+4\beta^2-24\beta-20\right)\rho_J^2+2\left(5\beta^5+29\beta^4+66\beta^3+70\beta^2+32\beta+4\right)\rho_J\right)\\
&+\rho_A^3\left(\beta+\rho_J+1\right)^2\left(\beta\left(3\beta^2+13\beta+12\right)(\beta+1)^3\right.\\
&\left.+\left(5\beta^4+20\beta^3+26\beta^2+8\beta-4\right)\rho_J^2+2\left(3\beta^5+21\beta^4+52\beta^3+56\beta^2+24\beta+2\right)\rho_J\right)\\
&+\beta(3\beta+4)\rho_A^2\left(\beta+\rho_J+1\right)^2\left((\beta+1)^4+2(\beta+3)(\beta+1)^2\rho_J+(\beta+2)^2\rho_J^2\right)\\
&+\beta(\beta+1)^2(3\beta+4)\rho_J^2\left(\beta+\rho_J+1\right)^2=0.
\end{aligned}
$$

The fact that $\beta$ depends only on $\rho_A$ and $\rho_J$ is not a trivial one: it says that the relative bias depends only on the relative pool size ratio and the relative flux distribution, and has nothing to do with the absolute scale of the pool sizes or fluxes; this is consistent with the results in the case of metabolite removal.

Figure S12 plots the functional dependence of $\beta$ on $\rho_A$ and $\rho_J$, from which several salient features stand out. First, $\beta$ is negative: this is consistent with the reasoning above on the underestimation of $J$ (Figure S11b). Second, $\beta$ is small (in magnitude) when $\rho_A$ is small: when the $A_1$ pool is much larger than $A_3$ pool, most of the fitting cost comes from $A_1$ (due to its larger scale) rather than $A_3$ and therefore the fitting becomes insensitive to the dynamics of $A_3^*$ which is the only thing changed in the reduced model (Figure S11b). Third, when $\rho_A$ exceeds around 0.5, $\beta$ shows a strong positive dependence on $\rho_J$: the bias gets more severe with the proportion of flux leaking to $A_2$, which makes intuitive sense as the more flux leaks to $A_2$, the more the reduced model deviates from the full one.

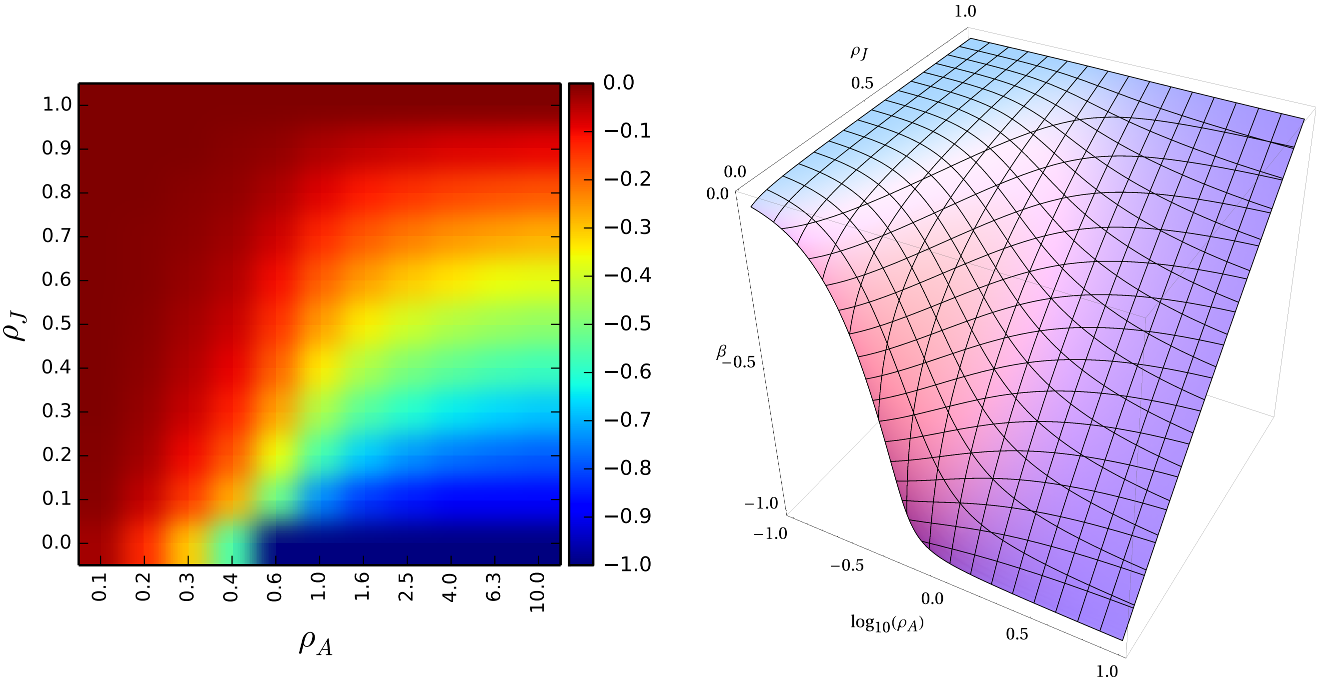


(a) Heatmap of $\beta$ as a function of $\rho_A$ and $\rho_J$.

(b) 3D-view of the same function.

Figure S12: How pathway removal affects KFP.

To sum up, for the pathway removal and KFP in Figure S11a, the bias in the estimated flux is small when the metabolite at the branch point dominates the other in pool size ($A_1 \gg A_3$) *or* most of the flux goes down the retained pathway rather than leaks to the removed one ($J_3 \gg J_2$). Below we will see how this result can also be used to understand the bias of pathway removal in rKFP.

### S3.2.2 rKFP

We last discuss how pathway removal affects rKFP. Figure S13a shows a diagram of the reduction. Using the same steps in the analytical approach again, where the cost is now an integral of four terms,

$$C = \int_0^\infty \left((\tilde{a}^*_{1x} - a^*_{1x})^2 + (\tilde{a}^*_{1y} - a^*_{1y})^2 + (\tilde{a}^*_{3x} - a^*_{3x})^2 + (\tilde{a}^*_{3y} - a^*_{3y})^2\right) \mathrm{d}t,$$

we find that in this final case the bias in the parameter of interest, the relative flux change $R_1 \equiv J_{1y}/J_{1x}$, has lost its previous simple mathematical structure and is now a function of six dimensionless variables: $\beta_R \equiv \frac{\tilde{R} - R_1}{R_1} = f(r_1, R_1, \rho_A, \rho_r, \rho_J, \rho_R)$, where $\tilde{R}$ is the estimated relative flux change $\frac{\tilde{J}_y}{\tilde{J}_x}$, $r_1$ is $A_1$'s relative pool size change $\frac{A_{1y}}{A_{1x}}$, $\rho_A$ is the relative pool size difference between the two metabolites in the control $\frac{A_{3x}}{A_{1x}}$, $\rho_r$ is the relative difference in the relative pool size changes between the two metabolites $\frac{r_3}{r_1}$ (note that the relative pool size difference between the two metabolites in the *condition* becomes $\frac{A_{3y}}{A_{1y}} = \frac{r_3 A_{3x}}{r_1 A_{1x}} = \rho_r \rho_A$), $\rho_J$ is the relative proportion of flux going to $A_3$ in the control $\frac{J_{3x}}{J_{1x}}$, and $\rho_R$ is the relative difference in the relative flux change between the two metabolites $\frac{R_3}{R_1}$ (similarly, the relative proportion of flux going to $A_3$ in the *condition* becomes $\rho_{Jy} = \frac{J_{3y}}{J_{1y}} = \frac{R_3 J_{3x}}{R_1 J_{1x}} = \rho_R \rho_J$).

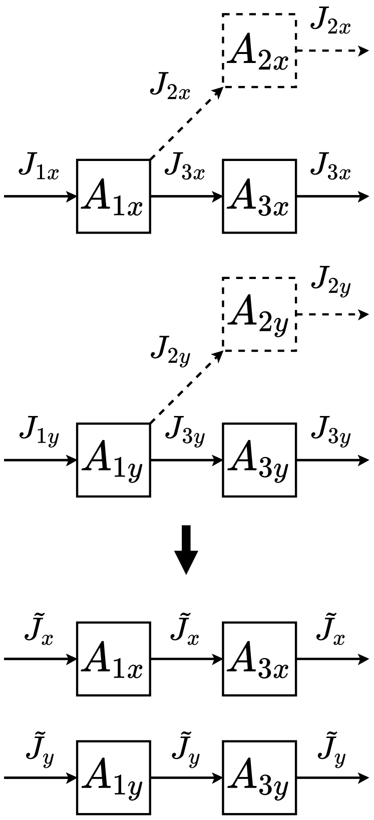


(a) Diagram of pathway removal in rKFP.

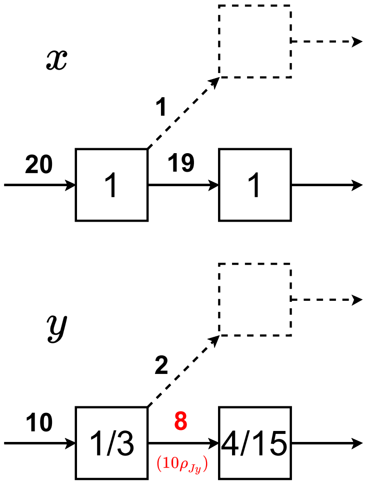


(b) The metabolite concentrations in the condition ($A_{1y}$ and $A_{3y}$) are calculated by using the common assumption that the reactions have Michaelis-Menten kinetics with Michaelis constants equal to the metabolite concentrations in the control ($A_{1x}$ and $A_{3x}$; *e.g.*, [14]). $\rho_{Jy}$ refers to the proportion of flux entering $A_3$ in condition.

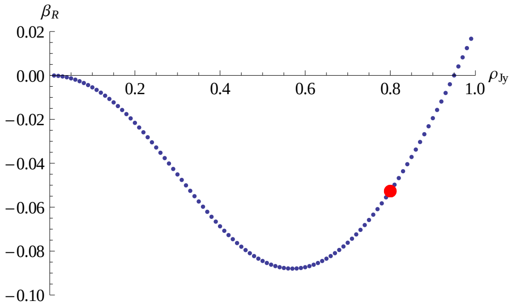


(c) Dependence of $\beta_R$ on $\rho_{Jy}$, where the red dot denotes the parameter choice in (b).

Figure S13: How pathway removal affects rKFP.

Plotting the dependence of $\beta_R$ on all six variables might be neither convenient nor illuminating, hence we seek other means to glean insight. The first thing we verify is that $\beta_R = 0$ when $\rho_R = 1$ regardless of the values of other variables. This is important: when the pathway fluxes change in same manner, there is no bias in the estimated relative flux change $R$, which is in spirit very similar to the case of metabolite removal (certainly as a consequence of some kind of canceling mechanism that is unfortunately hard to unravel altogether here due to the complexity).

However, while in the case of metabolite removal we have both theoretical expectation and empirical observation that metabolites along the same pathway tend to change in a similar fashion and indeed cancel much of the bias in rKFP estimations (see main text), in this case we have no *a priori* reasons to believe that pathways also change similarly between two conditions. In fact, due to the decision-point nature of a branch point where multiple branching pathways emanate (Figure S6), fluxes at a branch point would likely respond differently to perturbations if the perturbations cause a switch in metabolic decisions. Therefore, we need to investigate the dependence of $\beta_R$ on deviations from perfect co-response of the pathways.

The first conclusion along this line is that if the pathway retained in the reduced model is the major one in both conditions (*i.e.*, both $\rho_{Jx} = J_{3x}/J_{1x}$ and $\rho_{Jy} = J_{3y}/J_{1y}$ are close to one), $\rho_R$ would still be close to one even when there is significant metabolic reprogramming. Figure S13b shows a numerical example of this: when a branch point changes the flux configuration from (20,1,19) to (10,2,8), the metabolic decision has changed ($J_2$ pathway upregulated by 100% compared to others being downregulated by 50% or more), but $\rho_R = \dfrac{R_3}{R_1} = \dfrac{8/19}{10/20} = \dfrac{16}{19} \approx 0.84$, resulting in a roughly 5% bias of the estimated $R$ in our numerical example. Alternatively, using the idea of "decomposition"

described in Section S3.1.2 handwavingly, one can think that when both $\rho_{Jx}$ and $\rho_{Jy}$ are close to one, the result in Section S3.2.1 says that the biases introduced individually to the flux estimates of the two conditions are small, hence making the overall bias of $R$ in rKFP small.

Second, to assess the scenarios where the major branching pathway has switched (*e.g.*, $\rho_{Jx} = J_{3x}/J_{1x}$ is close to one while $\rho_{Jy} = J_{3y}/J_{1y}$ is close to zero), we use the numerical example in Figure S13b and move the major branching pathway gradually from the $J_3$ pathway to $J_2$ pathway. One has to be careful in doing this: as mentioned above $\beta_R$ also depends on other variables such as $\rho_r = r_3/r_1$, and changing the major branching pathway from $J_3$ to $J_2$ (*i.e.*, decreasing $\rho_{Jy}$ in Figure S13b) entails changes in the concentrations of the metabolites as well through the laws of enzyme kinetics, which in turn changes $\rho_r$. For the numerical example, if $\rho_{Jy}$ is decreased from 0.8 to 0.2, then according the computation model described in the caption of Figure S13b, $A_{3y}$ will no longer be 4/15, but rather 1/18, since the laws of enzyme kinetics requires a lower concentration to match the lower flux. Taking this concentration dependence into account, we calculate $\beta_R$ as a function of $\rho_{Jy}$ (Figure S13c). Rather surprisingly, $\beta_R$ is well contained within 9% and actually drops to zero even when the branch point has totally switched to $J_2$. To explain this, we need to invoke the other condition for a small $\beta_J$ in KFP (Section S3.2.1): $A_1 \gg A_3$. When the $J_1$ flux is simply diverted from $J_3$ to $J_2$, $A_3$ drops but $A_1$ remains the same, which brings down $\beta_J$; using the decomposition idea again, $\beta_J$ is small in control because $J_3 \gg J_2$, and is small in condition because $A_1 \gg A_3$, hence the small $\beta_R$.

To sum up, the bias in the estimated relative flux change is small when pathways change in the same manner ($R_1 \approx R_3$), or when the retained pathway is the major one in both conditions ($J_{3x} \gg J_{2x}$ and $J_{3y} \gg J_{2y}$); even if these two fail to hold, we are still guarded by enzyme kinetics which keeps the bias small.

### S3.3 How Lumping Isomers Affects KFP

The main text has discussed how metabolite removal affects KFP, and in this section we provide some results and intuitions on the effects of lumping isomers on KFP. First, lumping serial metabolites (Figure S14a): both bias (Figure S14b) and error ratio (Figure S14c) are symmetric in $q$ with respect to $q = 0.5$, which has its root in the mathematical structure of $^{13}C$ moving along a pathway (see Remark 4 in the previous section); the underestimation of $J$ that grows as $q$ tends to 0.5 can be explained by the slowdown of $^{13}C$ labels along the pathway by a serial bipartition of a metabolite pool that peaks at equal bipartition (Figure S17a); error ratio decreases when $A$ increases, suggesting that the reward of distinguishing the isomers grows with their pool size.

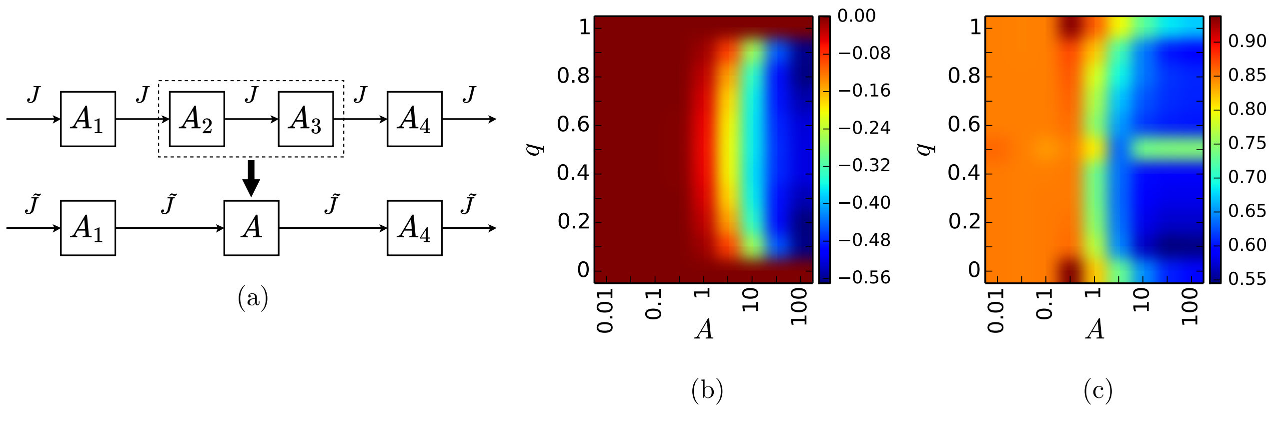


Figure S14: Lumping of serial metabolites in KFP. (a) Diagram. Parameter values and parameterization used for generating the simulated data: $A_1 = A_4 = J = 1$, $A_2 = Aq$, $A_3 = A(1-q)$. (b) Bias. (c) Error ratio.

Second, lumping parallel metabolites: bias (Figure S15c) is now antisymmetric in $q$ with respect to $q = 0.5$, which can be explained by a quasi-antisymmetric effect of a parallel bipartition on the infiltration dynamics along a pathway as shown in Figure S17b.

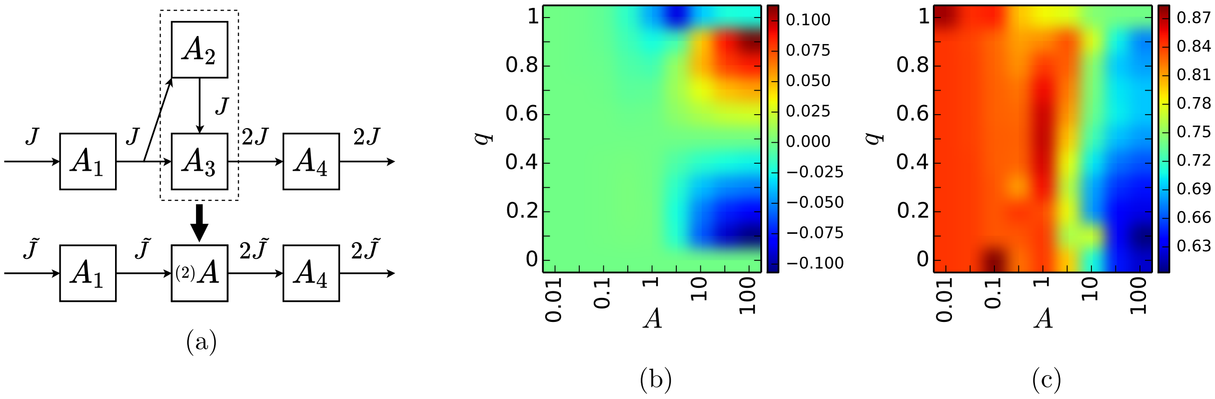


Figure S15: Lumping of parallel metabolites in KFP. (a) Diagram. The scenario is modeled on the reactions of ALD and TPI in glycolysis, hence the doubling of flux in the lower half. To keep the stoichiometry comparable, we set that one $A_1$ forms two $A$ and one $A$ forms one $A_4$ in the reduced network. Parameter values and parameterization used for generating the simulated data: $A_1 = A_4 = J = 1,\ A_2 = Aq,\ A_3 = A(1-q)$. (b) Bias. (c) Error ratio.

One important cautionary note: here all reactions are assumed irreversible; however, as discussed at the end of the section on modeling reversible reactions, most isomers are close to equilibrium, and therefore lumping them into a single pool is probably much more innocuous than presented here; when non-isomers are lumped, the results here become more relevant.

## S3.4 Plots of costs

The plots of the costs in three cases are provided below.

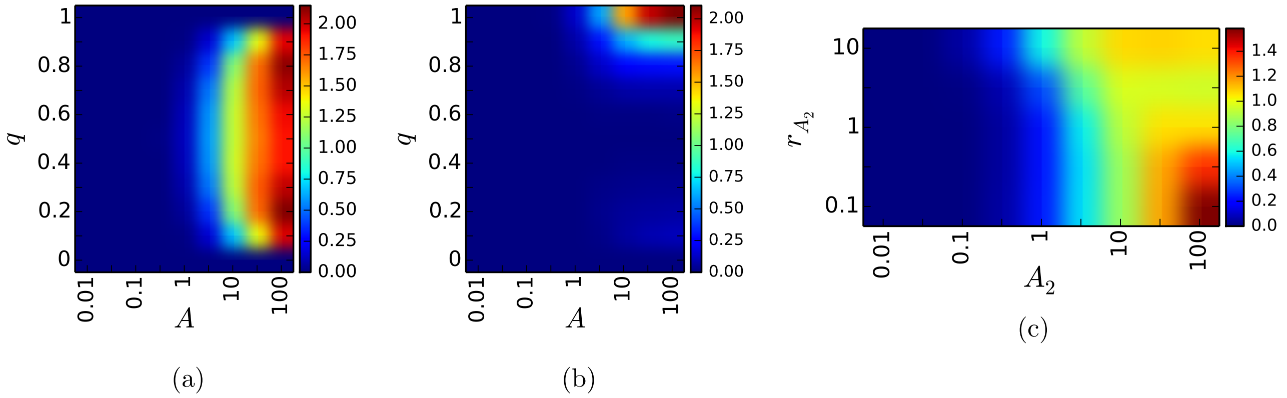


Figure S16: Costs of the three cases. (a) KFP in lumping of serial metabolites. (b) KFP in lumping of parallel metabolites. (c) rKFP in metabolite removal.

## S3.5 Dynamics of serial and parallel bipartition

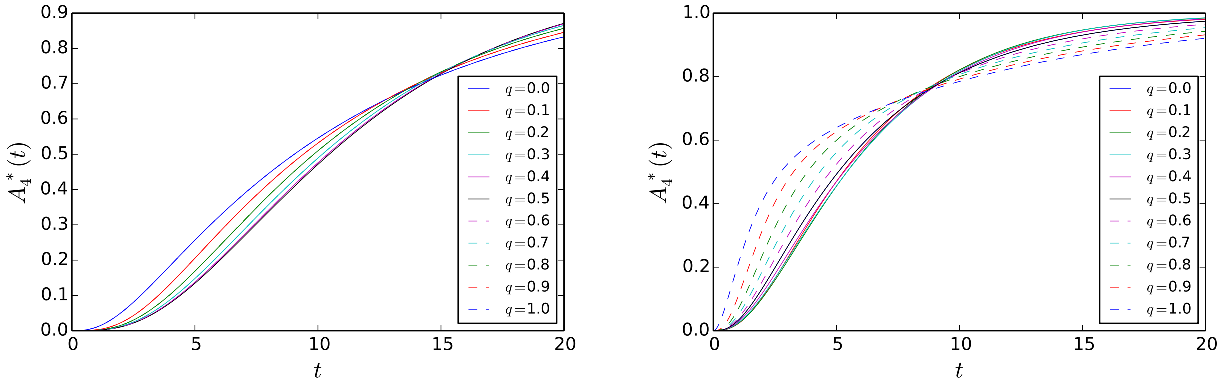


(a) (b)

Figure S17: Understanding the effects of serial and parallel bipartitions on the dynamics of the pathway. Parameter values used in generating the simulated data: $A_1 = A_4 = J = 1$ and $A = A_2 + A_3 = 10$. (a) Dynamics of $A_4^*(t)$ in KFP applied to lumping of serial metabolites (Figure S14a). It shows that a serial bipartition of a metabolite pool slows down $^{13}$C labels and the slowdown peaks at equal bipartition $q = 0.5$. That the solid curves overlap with the dashed curves shows again the symmetry of $A_4^*(t)$ in $q$ about $q = 0.5$ (Remark 4 in the previous section; Figs S14b & S14c). (b) Dynamics of $A_4^*(t)$ in KFP applied to lumping of parallel metabolites (Figure S15b). It shows that a parallel bipartition of a metabolite pool affects the infiltration of $^{13}$C labels in a complex way: as $q$ increases from 0 to 0.5 and the bipartition changes from a concentration in $A_3$ to an equal one, it first slows down the dynamics, reaching the slowest around $q = 0.2$, and then comes back to its starting point (the black curve overlaps with the blue one); as $q$ increases from 0.5 to 1 and the bipartition changes from an equal one to a concentration in $A_2$, it short-circuits the pool and speeds up the dynamics. In short, its effect is roughly antisymmetric about $q = 0.5$: slowing things down when $q < 0.5$ and speeding things up when $q > 0.5$.

## S3.6 Summary Table

The tables below summarize the results of using reduced models, give general practical suggestions, and contain pointers to the relevant details.

Table S1: effects of different reduction scenarios (MR: metabolite removal; PR: pathway removal; L: lumping; AI: assuming irreversibility)

| Scenario | Effects | Full or Reduced Model in General | Result Reference |
|---|---|---|---|
| MR in KFP | underestimation; worsens as missing pool size gets large | Full | Figures 2, S9 |
| MR in rKFP | worsens as missing pool size gets large or changes differently | Full | Figures 3, S10 |
| PR in KFP | underestimation; worsens as missing pathway gets major or pool size at the retained pathway gets large | Full | Figures S11, S12 |
| PR in rKFP | usually mild | Reduced | Figures S13 |
| L in KFP | usually underestimation; usually harmless | Reduced | Figures S17a , S17b |
| L in rKFP | usually harmless | Reduced | |
| AI in KFP | underestimation; worsens as it gets reversible | Reduced | Figure 4 |
| AI in rKFP | worsens as pool size changes differ | Reduced | Figure 4 |

Table S2: Detailed summary of the effects of metabolite removal and lumping.

| | KFP | rKFP |
|---|---|---|
| Metabolite removal | Underestimates $J$; worsens as the removed pool size increases; error ratio roughly constant at 0.35 (Figure 2b) | Under-/over-estimates $r_J$ when the removed pool size increases more/less than others; worsens as the pool size increases; likely *robust* due to canceling; error ratio decreases as the pool size or its change ratio increase (Figures 3c & S16c) |
| Lumping of serial metabolites | Underestimates $J$; underestimation worsens as $q$ approaches 0.5 and the lumped pool size increase; error ratio decreases similarly (Figures S17a & S16a) | |
| Lumping of parallel metabolites | Underestimates $J$ when $q < 0.5$ and overestimates when $q > 0.5$; worsens as the lumped pool size increases; error ratio decreases similarly (Figures S17b & S16b) | |

# S4 Selecting Measuring Times

This section discusses the selection of measuring times for (r)KFP, and we believe that they shed light on both the experimental design and conceptual underpinning of (r)KFP.

As in Methods we have shown how errors of the estimates depend on Jacobian $\mathbf{D}$ and how $\mathbf{D}$ depends on measuring times, one can formulate the problem as the following: Given a model with one parameter $\theta$ of great inferential interest, find a collection of measuring times $\mathbf{t} = (t_1, ..., t_l)$ such that the error of $\theta$, $\sigma_\theta(\mathbf{t})$, is the smallest. $\theta$ would be $J$ in KFP or $r_J$ in rKFP; if more than one parameter is of interest, the quantity to be minimized can be the product of their errors, $\prod_\alpha \sigma_{\theta_\alpha}$.

The above formulation translates the experimental design problem to an optimization one, leaving little more to be said than the routine application of optimization algorithms. However, such an approach is unsatisfying for three reasons. First, the optimization problem is computationally nontrivial: it is nonlinear, nonconvex (Figure S19), and has constraints as the measuring times are nonnegative and bounded; furthermore, $\sigma_\theta$ is invariant to a reordering of times but using this symmetry in the optimization is challenging. Second, the formulation requires detailed knowledge of parameter values, which is unrealistic in designing real experiments where the aim is exactly to estimate the parameter values. Third, the optimal sampling times found in this way can be hard to interpret physically.

Assuming having a rough prior guess, but not detailed knowledge, of the parameter values, we modify our aim to be using the prior information and having a sensible selection of the measuring times. We also adopt a more physical kind of reasoning for the purpose: since different labeled metabolites rise up with their own time-scales (Figure S4b), a natural selection would be to have different measuring times capture the dynamics of different metabolites. Below we describe our reasonings in some details using the example of applying KFP to a linear pathway with irreversible reactions (Figure S4a).

We first note that one natural measuring time would be a late one, one that is in the constant regime of all metabolites, so that the scales of all metabolites can be estimated at once. With the scale information, now consider the first metabolite $A_1$. For pedagogical purpose, we first assume that $A_1$ is known, equivalent to the late time being measured many times and the scale estimated with great precision, and that one more time can be measured. That is, for the curve $A_1^*(t) = A_1(1 - e^{-\frac{Jt}{A_1}})$ with $A_1$ known, find a time $t$ to minimize $\sigma_J$. With only one parameter $J$ and one measurement $A_1^*(t)$, the Jacobian $\mathbf{D}$ is the scalar $\dfrac{dA_1^*}{dJ}$, and, assuming constant noise, $\sigma_J$ is proportional to its inverse: $\sigma_J \propto \left(\dfrac{dA_1^*}{dJ}\right)^{-1}$ (Methods). Since $\dfrac{dA_1^*}{dJ} = te^{-\frac{Jt}{A_1}}$, we find its maximum by setting its time derivative to 0: $\dfrac{\partial}{\partial t}\dfrac{dA_1^*}{dJ} = e^{-\frac{Jt}{A_1}}\left(1 - \dfrac{Jt}{A_1}\right) = 0$, which gives us $t = \dfrac{A_1}{J} = t_c$, the characteristic time-scale. To sum up, if $A_1$ is known and only one time is measured, one should measure at $t_c$ to minimize the error in $J$. This gives rise to one more significance of $t_c$ (Figure 1b).

We next relax the assumption of known $A_1$, and pose the following problem: for the curve of $A_1^*(t)$ with both $A_1$ and $J$ unknown and given two measuring times, if one is at a late time, find the other time $t$ to minimize $\sigma_J$. Now with two measurements and two parameters, the Jacobian $\mathbf{D}$ is a 2-by-2 matrix, and getting the form of $\sigma_J$ as a function of $t$ is more involved. After some calculations (details in Section S4.4), we find that $t \approx 0.92\ t_c$. In other words, if $A_1$ is unknown but a late time measured, one should measure at $0.92\ t_c$ to minimize the error in $J$, slightly earlier than when $A_1$ is known.

What if $A_1$ is unknown but a late time measured, and two more times, $t_1$ and $t_2$, can be measured? Figure S20a shows $\sigma_J$ as a function of $t_1$ and $t_2$, which gives that $\sigma_J$ is minimal when $t_1 = t_2 = 0.87\ t_c$. That $t_1 = t_2$ might be surprising as it seems redundant to have both measurements at the same time; however, note that measurement noise decreases with the square-root of the number of measurements, and hence it can be intuitively explained that $0.87\ t_c$ is so much more informative than any other times that one should get one noise-reduced measurement at $0.87\ t_c$ rather than two full-noise measurements at two other times. A similar observation can be made when three more times are to be measured. Figure S20b summarizes all the cases, and shows that although the optimal measuring time gradually deviates from $t_c$ as the number of measurements increases, $\sigma_J$ at $t_c$ is close to the true minimal $\sigma_J$.

Since $t_c$ is close to optimal and easy to interpret, we therefore provide the following simple suggestion for choosing the measuring times for $A_1$ given some estimate of $A_1$ and $J$: after measuring at a late time, other measurements should be placed around $A_1/J$.

Having understood the case of $A_1$, we can apply a similar reasoning to the second metabolite $A_2$ at a much more brisk pace. For the curve $A_2^*(t)$ (Eq. S7), we first assume $A_1$ and $A_2$ to be known, and solve for a single time $t$ that minimizes $\sigma_J$: assuming $A_1 \approx A_2$, we find that $t = (A_1 + A_2)/J$, the sum of $t_c$ for $A_1$ and $A_2$ (details in Section S4.5). This makes intuitive sense, given that $A_2^*(t)$ is a mixture of two time-scales due to the actions of both $A_1$ and $A_2$ pools (Section S1). Simulation results confirm that when the assumptions of known $A_1$ and $A_2$ and $A_1 \approx A_2$ are relaxed and more times are measured, $t = (A_1 + A_2)/J$ is still close to optimal, like the case of $A_1$ (Figure S21).

Applying the same reasoning to the k-th metabolite $A_k$ gives similar results, and we therefore provide the following general suggestion for a linear pathway of k metabolites given some estimate of the pool sizes and flux: after measuring at a late time, other measurements should be placed around $\sum_{i=1}^{k} A_i/J$, $k = 1, 2, \ldots$. A final remark comes from the observation that metabolites in a typical metabolic network have strong separation of concentration scales. For the example of glycolysis, one source reports that the most abundant metabolite (glucose) is 35 times more than the second most abundant one [4]. This has an important implication: As can be seen from $t = \sum_{i=1}^{k} A_i/J$, any $A_i$ greatly larger than others will dominate the numerator, effectively compressing all time-scales into one (see also Section S1). Therefore, all metabolites downstream of a dominant one share the dynamics and measuring times with it, and glucose being both the most abundant and first metabolite in glycolysis suggests that the whole pathway has effectively only one time-scale, greatly simplifying our understanding and practice for the system.

## S4.1 Integrating Sensitivity Curves

Here we explain how sensitivity curves are calculated in SloppyCell (Methods). For a system of ODEs $\frac{dA_i^*}{dt} = f(\mathbf{A}^*, \boldsymbol{\theta})$ $(i = 1, ..., n)$, the parameter sensitivity of $A_i^*$, $\frac{\partial A_i^*}{\partial \theta_\alpha}$ is a function of time, and has the following rate of change according to the chain rule: $\frac{d}{dt}\frac{\partial A_i^*}{\partial \theta_\alpha} = \frac{\partial}{\partial \theta_\alpha}\frac{dA_i^*}{dt} = \frac{\partial f}{\partial \theta_\alpha} + \sum_j \frac{\partial f}{\partial A_j^*}\frac{\partial A_j^*}{\partial \theta_\alpha}$, which constitutes another system of ODEs, now in $mn$ dimension with $\frac{\partial A_i^*}{\partial \theta_\alpha}$ as the variables. SloppyCell calculates the right-hand side of new system of ODEs through analytical differentiation, and numerically integrates it to get $\frac{\partial A_i^*}{\partial \theta_\alpha}(t)$ [15].

Figure S18 shows an exemplary set of the sensitivity curves.

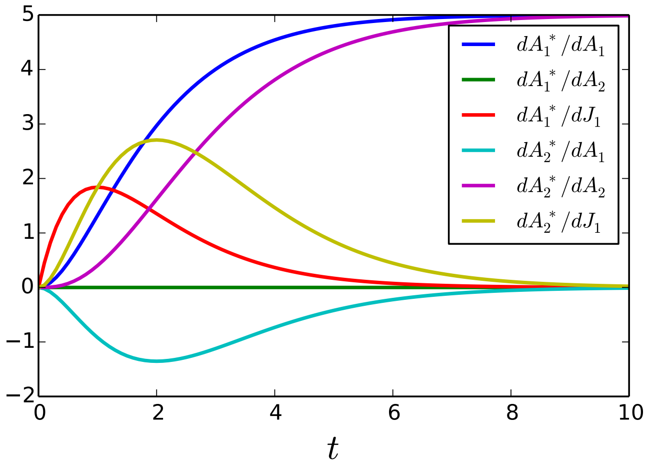


Figure S18: Some example sensitivity curves. The network is a linear pathway of two metabolites (Figure S4a) with parameters $A_1 = A_2 = J = 1$.

## S4.2 Nonconvexity of Minimizing $\sigma_J$

Here we show an example of the nonconvexity of the minimization problem of $\sigma_J$, hence the difficulty of searching for the global minimum.

Figure S19 shows the landscape of $\sigma_J$ on a single $t$ for the sensitivity curves in Figure S18 when measuring times of 1, 2, 3 and 100 are already chosen. That it has two local minima shows its nonconvexity; that one local minimum is around the mixed regime of $A_1$ and another local minimum around the constant regime reflects that measurements most informative of $A_i$'s are in the constant regime and measurements most informative of $J$ when $A_i$'s are known are in the mixed regime, and one can either go for the mixed regime and $J$ directly or the constant regime and $A_i$'s and hence $J$ indirectly.

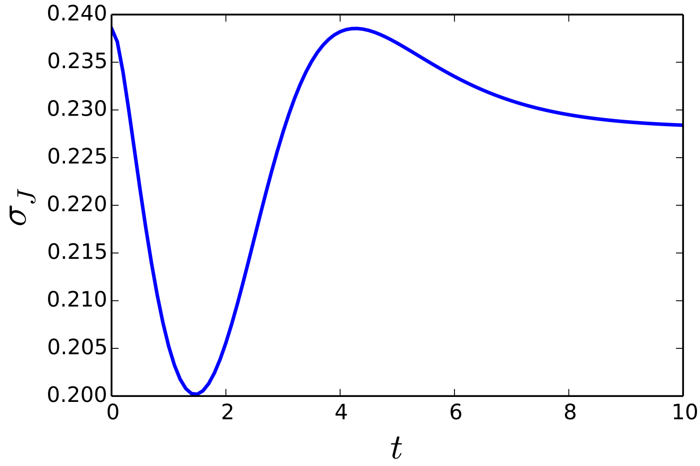


Figure S19: Nonconvex minimization landscape of $\sigma_J$.

## S4.3 The measuring times for $A_1$

Figures S20a and S20b are generated for the first metabolite in a pathway with parameters $A_1 = J = 1$.

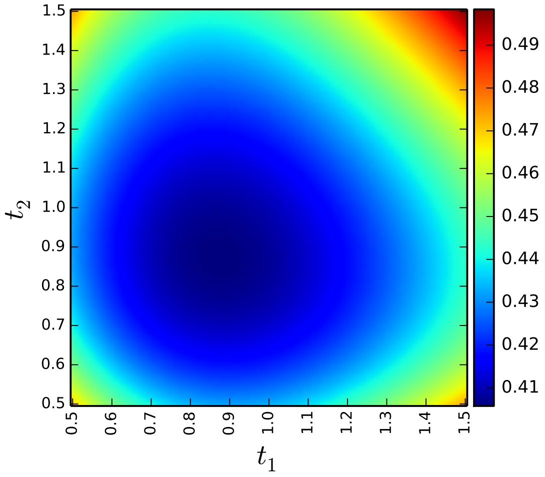


(a)

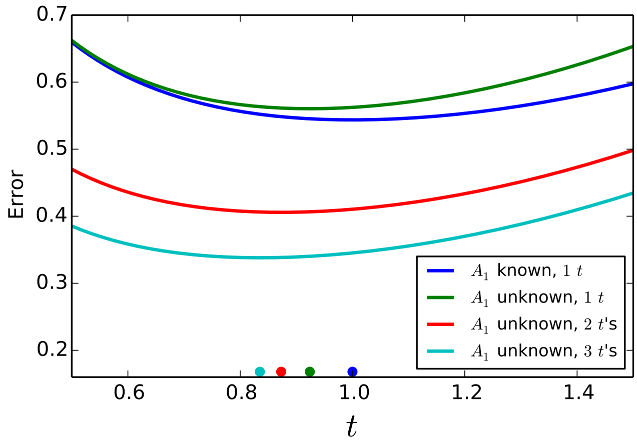


(b)

Figure S20: (a) The minimization landscape of $\sigma_J$ on two measuring times given one measurement going to the constant regime. Note that $\sigma_J$ is minimal when $t_1$ and $t_2$ are equal and smaller than 0.92 $t_c$ (see the main text). (b) The minimization landscape of $\sigma_J$ or its cross sections for different cases of measuring times (when $A_1$ is unknown, one measurement goes to the constant regime to estimate it). Note that the optimal $t$ shifts leftward away from $t_c = 1$ as $A_1$ becomes unknown and as more measurements are made (see the main text).

### S4.4 Calculating $t \approx 0.92\, t_c$

For $A^*(t) = A(1 - e^{-\frac{Jt}{A}})$ with both $A$ and $J$ unknown and two measurements at $t_1$ and $t_2$, the Jacobian $\mathbf{D}$ takes the following form:

$$\mathbf{D} = \begin{pmatrix} \dfrac{\partial A^*(t_1)}{\partial A} & \dfrac{\partial A^*(t_1)}{\partial J} \\ \dfrac{\partial A^*(t_2)}{\partial A} & \dfrac{\partial A^*(t_2)}{\partial J} \end{pmatrix}.$$

Plugging $\dfrac{\partial A^*(t)}{\partial A} = 1 - e^{-\frac{Jt}{A}} - e^{-\frac{Jt}{A}}\dfrac{Jt}{A}$, $\dfrac{\partial A^*(t)}{\partial J} = e^{-\frac{Jt}{A}}t$ and $t_2 = \infty$ (one measurement in the constant regime), $\mathbf{D}$ becomes $\begin{pmatrix} 1 - e^{-\frac{Jt_1}{A}} - e^{-\frac{Jt_1}{A}}\frac{Jt_1}{A} & e^{-\frac{Jt_1}{A}}t_1 \\ 1 & 0 \end{pmatrix}$. Now following the formula in Methods, to get $\sigma_J$ as a function of $t_1$ we need to get $\mathbf{\Sigma}$ and $\mathbf{V}$ in the singular value decomposition of $\mathbf{D}$ (assuming constant noise, we do not have to normalize the entries of $\mathbf{D}$ by the associated noise as the resulting $\sigma_J$ will only be shifted by a constant scale factor without affecting where it takes the minimum). Knowing that entries in $\mathbf{\Sigma}$, the singular values of $\mathbf{D}$, are the square roots of the eigenvalues of $\mathbf{D}^T\mathbf{D}$, and that $\mathbf{V}$ consists of normalized eigenvectors of $\mathbf{D}^T\mathbf{D}$, we perform eigendecomposition on $\mathbf{D}^T\mathbf{D}$ using *Mathematica*'s symbolic function `Eigensystem`: $\mathbf{D}^T\mathbf{D} = \mathbf{V}\mathbf{\Lambda}\mathbf{V}^{-1}$ (note that Mathematica does not normalize $\mathbf{V}$, but it does not matter for the same reason of not having to normalize $\mathbf{D}$ by the noise). The second diagonal entry of $\mathbf{V}\mathbf{\Lambda}^{-1}\mathbf{V}^T$ gives $\sigma_J$:

$$\sigma_J(t_1) = \frac{J}{A}\left(\frac{Jt_1}{A} - 2e^{\frac{Jt_1}{A}} + 2\right) + 1, \tag{S10}$$

which, when differentiated with respect to $t_1$ and set to zero, gives:

$$\left(\frac{Jt}{A_1} + 1\right) + e^{\frac{Jt}{A_1}}\left(\left(\frac{Jt}{A_1}\right)^2 - 2\right) - 2e^{\frac{2Jt}{A_1}}\left(\frac{Jt}{A_1} - 1\right) = 0 \tag{S11}$$

The transcendental equation has no analytical solution, and numerically we find $t \approx 0.92\, t_c$.

Note: the units in Eq. S10 do not match because the second diagonal entry in $\mathbf{D}$ has the unit of time but its value of 0 conceals it, and $\mathbf{D}$ was not normalized by the noise; the lack of matching in unit does not, however, affect the numerical values which are of concern here.

### S4.5 Deriving $t = (A_1 + A_2)/J$ for $A_2$

Given $A_2^*(t) = A_2\left(1 - \left(\dfrac{A_1}{A_1 - A_2}e^{-\frac{Jt}{A_1}} + \dfrac{-A_2}{A_1 - A_2}e^{-\frac{Jt}{A_2}}\right)\right)$ (Eq. S7) and ssuming $A_1$ and $A_2$ as known, we first try to solve for a formula of $t$ at which $\sigma_J$ is minimal in the same way as in $A_1$:

$$\begin{aligned}
\frac{\partial A_2^*(t)}{\partial J} &= -\frac{A_2 A_1}{A_1 - A_2}e^{-\frac{Jt}{A_1}}\left(-\frac{t}{A_1}\right) + \frac{A_2 A_2}{A_1 - A_2}e^{-\frac{Jt}{A_2}}\left(-\frac{t}{A_2}\right) \\
&= \frac{A_2}{A_1 - A_2}t\left(e^{-\frac{Jt}{A_1}} - e^{-\frac{Jt}{A_2}}\right) \\
\frac{\partial}{\partial t}\frac{\partial A_2^*(t)}{\partial J} &= \frac{A_2}{A_1 - A_2}\left(\left(e^{-\frac{Jt}{A_1}} - e^{-\frac{Jt}{A_2}}\right) + t\left(e^{-\frac{Jt}{A_1}}\left(-\frac{J}{A_1}\right) - e^{-\frac{Jt}{A_2}}\left(-\frac{J}{A_2}\right)\right)\right) = 0,
\end{aligned}$$

which simplifies to:

$$e^{-\frac{Jt}{A_1}} - e^{-\frac{Jt}{A_2}} = \frac{Jt}{A_1}e^{-\frac{Jt}{A_1}} - \frac{Jt}{A_2}e^{-\frac{Jt}{A_2}}, \tag{S12}$$

a transcendental equation in $t$ without an analytical solution. We apply perturbation analysis to it to get an approximate solution of $t$ valid when $A_1$ and $A_2$ are close. Assuming $\dfrac{A_1}{A_2} = 1 + \epsilon$ and $\epsilon \ll 1$, we replaced $A_1$ in Eq. S12 by $(1 + \epsilon)A_2$:

$$e^{-\frac{Jt}{(1+\epsilon)A_2}} - e^{-\frac{Jt}{A_2}} = \frac{Jt}{(1+\epsilon)A_2} e^{-\frac{Jt}{(1+\epsilon)A_2}} - \frac{Jt}{A_2} e^{-\frac{Jt}{A_2}},$$

which, when letting $\tau \equiv Jt/A_2 = \mu_2 t = t/t_{2c}$ where $t_{2c}$ is the characteristic time-scale for $A_2$, becomes:

$$e^{\frac{\epsilon\tau}{1+\epsilon}} = \frac{1-\tau}{1-\frac{\tau}{1+\epsilon}}, \tag{S13}$$

an equation that specifies $\tau$ as a function of $\epsilon$. To get an approximate analytical expression of this function, we make use of the assumption that $\epsilon \ll 1$ and approximate the left-hand side by truncating its Taylor expansion at $\epsilon = 0$ after a few terms:

$$e^{\frac{\epsilon\tau}{1+\epsilon}} = 1 + \frac{\epsilon\tau}{1+\epsilon} + \frac{1}{2}\left(\frac{\epsilon\tau}{1+\epsilon}\right)^2 + \ldots$$

If we keep only the first two terms, Eq. S13 gives the following solution: $\tau = 2(1+\epsilon)$, equivalent to $t = 2A_1/J$; if we keep the first three terms, however, Eq. S13 gives the following solution: $\tau = \frac{(1+\epsilon)\sqrt{4+12\epsilon+\epsilon^2} - 2 - \epsilon + \epsilon^2}{2\epsilon}$, which, after linearization, becomes $\tau = 2+\epsilon$, equivalent to $t = (A_1+A_2)/J$. This agrees with analysis using perturbation series.

### S4.6 Close-to-optimality of $(A_1 + A_2)/J$ for $A_2$

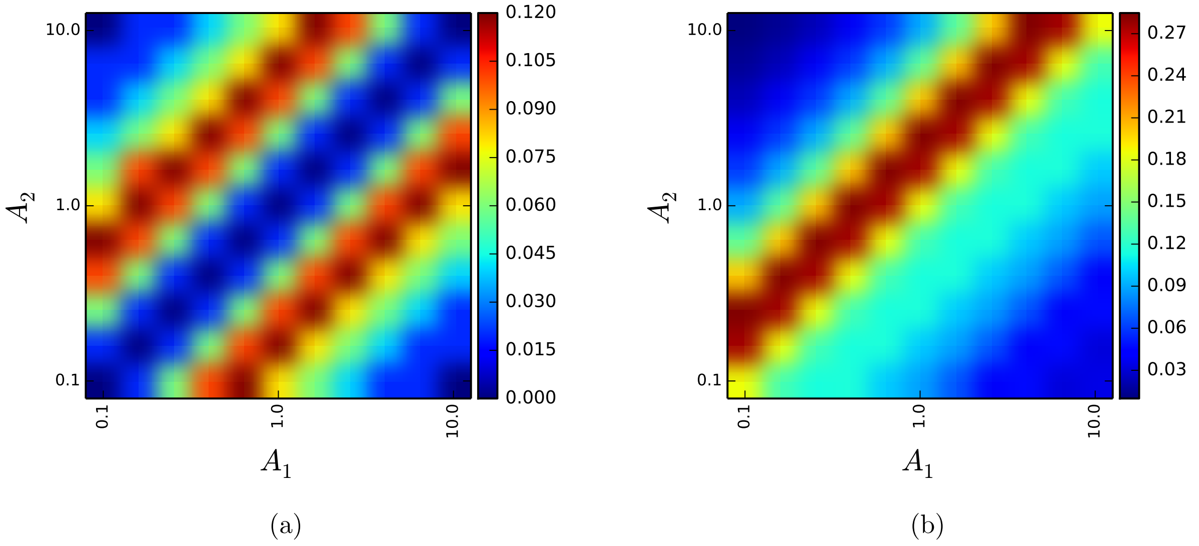


Figure S21: The normalized difference between the analytical result of $(A_1 + A_2)/J$ assuming $\epsilon \ll 1$ and the computational results of optimal $t$: $\frac{(A_1+A_2)/J - t}{t}$, using parameter value $J = 1$. (a) Assume that $A_1$ and $A_2$ are known, and one measurement is to be made; the approximation is good when $A_1 \approx A_2$ as expected, and also when $A_1 \gg A_2$ or $A_1 \ll A_2$ as there is only one effective rate in this case. (b) Assume that $A_1$ and $A_2$ are unknown, two measurements already made, one in the constant regime and one at $A_1/J$, and one more measurement is to be made.

### S4.7 Dependence of Estimation Precision on Data Size and Time Selection

This section lists some results on an important practical matter: suppose we want to estimate a flux $J$ to some given precision, how many data points do we need and how should they be assigned on different time-scales corresponding to different metabolites?

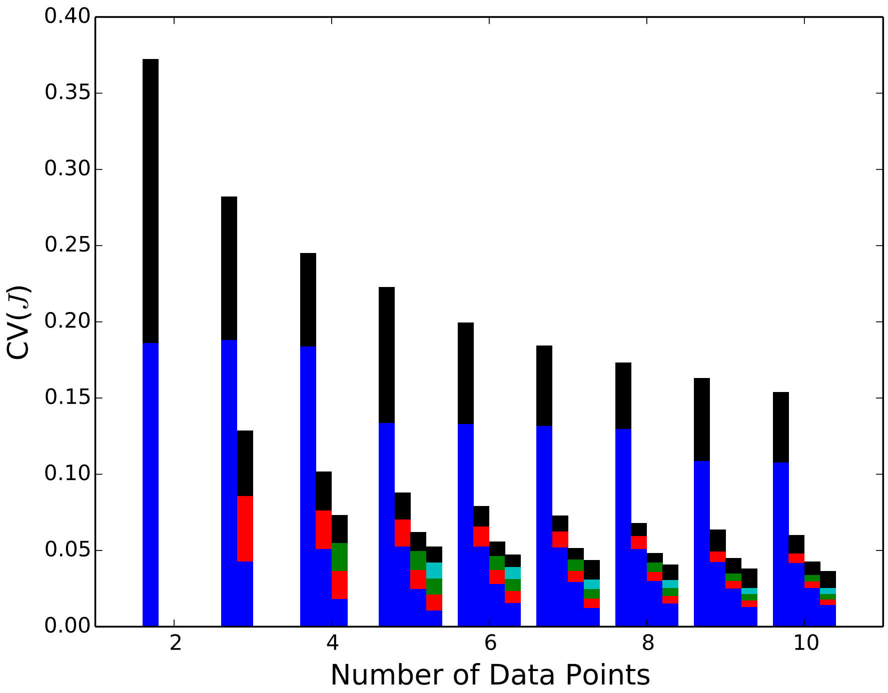


Figure S22: Estimation precision of $J$ as a function of data size and measuring times for linear pathways. The height of bars corresponds to the *coefficient of variation* (CV) of parameter $J$, defined as the estimation error divided by the estimated value. Each group of up to four bars corresponds to a data size, that is the number of data points one can afford to collect. Within each group individual bars correspond to linear pathways of one metabolite on the left, up to four metabolites on the right (a linear pathway of, say, four metabolites has five different time-scales, one for each metabolite and one for a late time in the constant regime, and requires at least five data points to cover them all, hence the less-than-four bars in a group when the data size is small). The color division within each bar corresponds to how to optimally assign the measuring times; using the example of a linear pathway of four metabolites again, the height of each of the five colors corresponds to the relative measuring density of that time-scale (for example, when ten data points can be afforded, they should be placed on the five time-scales with a [4,1,1,1,3] scheme, the first time-scale measured four times, the constant regime measured three times, etc.). We assume a 20% noise for each measurement, in accordance with the typical experimental resolution of mass spectrometry, therefore placing more measurements on important time-scales helps reduce the noise of the measurements at the time-scale (which typically scales inversely with the square root of the number of measurements) and achieve overall better estimation precision. The color scheme is chosen in such a way that blue always corresponds to the first time-scale, and black always corresponds to the constant regime, which makes it more apparent that the first and last time-scales are important and hence favored. Parameter values used for the simulation: all metabolite pool sizes and the flux are set to one.

Figure S22 plots such a relationship using simulation results on a linear pathway, and conveys a few key messages. First, the estimation of $J$ is much better off when one measures more than one metabolite in a pathway; in principle one can concentrate all the measurements on the first metabolite of the pathway to estimate $J$ (Figure 1 in the main text), but the figure says that it is better to spread them out on several metabolites in the pathway, even though additional metabolites introduce additional parameters (pool sizes); it also says that the gain decreases quickly with the number of metabolites beyond two. Second, the estimation precision increases with the number of measurements as expected. Third, the first and last time-scales, corresponding to the first metabolite and the constant regime, are heavily favored in the time selection, meaning that they are more informative than other time-scales in terms of estimating $J$ and one should place most of the measurements around them so that the dynamics around these time-scales can be measured with high precision; intuitively, the first time-scale is important because it affects the dynamics of all metabolites in the

pathway, and the last one is important because it allows us to estimate the scales (total concentration or signal) of all metabolites at once. Numerically, the figure also tells us that in order to estimate $J$ within, say, 5%, one needs to make at least eight measurements on a few metabolites with a focus on the first and last time-scales.

# References


[1] Voet, D. (2011). Fundamentals of Biochemistry. Life at the Molecular Level Voet. Judith G. Voet, Charlotte W. Pratt, Wiley, Chichester, UK.

[2] Boyce, W. E., & DiPrima, R. C. (2006). Elementary Differential Equations and Boundary Value Problems. John Wiley & Sons, New York.

[3] Hirsch, M. W., Smale, S., & Devaney, R. L. (2004). Differential equations, dynamical systems, and an introduction to chaos (Vol. 60). Academic press.

[4] Garrett, R., and Charles M. Grisham. 2010. Biochemistry. Belmont, CA: Brooks/Cole, Cengage Learning.

[5] Milo, R., Shen-Orr, S., Itzkovitz, S., Kashtan, N., Chklovskii, D., & Alon, U. (2002). Network motifs: simple building blocks of complex networks. Science, 298(5594), 824-827.

[6] Heijnen, J. J., Van Gulik, W. M., Shimizu, H., & Stephanopoulos, G. (2004). Metabolic flux control analysis of branch points: an improved approach to obtain flux control coefficients from large perturbation data. Metabolic engineering, 6(4), 391-400.

[7] Palsson, B. O. (2006). Systems biology. Cambridge university press.

[8] Mu, F., Williams, R. F., Unkefer, C. J., Unkefer, P. J., Faeder, J. R., & Hlavacek, W. S. (2007). Carbon-fate maps for metabolic reactions. Bioinformatics, 23(23), 3193-3199.

[9] Hrl, M., Schnidder, J., Sauer, U., & Zamboni, N. (2013). Nonstationary 13Cmetabolic flux ratio analysis. Biotechnology and bioengineering, 110(12), 3164-3176.

[10] Wiechert, W. (2001). $^{13}$C Metabolic Flux Analysis. Metabolic engineering, 3(3), 195-206.

[11] Szecowka, M., Heise, R., Tohge, T., Nunes-Nesi, A., Vosloh, D., Huege, J., ... & Arrivault, S. (2013). Metabolic fluxes in an illuminated Arabidopsis rosette. The Plant Cell Online, 25(2), 694-714.

[12] Antoniewicz, Maciek R., Joanne K. Kelleher, and Gregory Stephanopoulos. "Elementary metabolite units (EMU): a novel framework for modeling isotopic distributions." Metabolic engineering 9.1 (2007): 68-86.

[13] Munger, J., Bennett, B. D., Parikh, A., Feng, X. J., McArdle, J., Rabitz, H. A., ... & Rabinowitz, J. D. (2008). Systems-level metabolic flux profiling identifies fatty acid synthesis as a target for antiviral therapy. Nature biotechnology, 26(10), 1179-1186.

[14] Stanford, N. J., Lubitz, T., Smallbone, K., Klipp, E., Mendes, P., & Liebermeister, W. (2013). Systematic construction of kinetic models from genome-scale metabolic networks. PloS one, 8(11), e79195.

[15] Ryan N. Gutenkunst, Jordan C. Atlas, Fergal P. Casey, Brian C. Daniels, Robert S. Kuczenski, Joshua J. Waterfall, Chris R. Myers, & James P. Sethna. (2007) SloppyCell. http://sloppycell.sourceforge.net/
